# The global role of ppGpp synthesis in morphological differentiation and antibiotic production in *Streptomyces coelicolor *A3(2)

**DOI:** 10.1186/gb-2007-8-8-r161

**Published:** 2007-08-03

**Authors:** Andrew Hesketh, Wenqiong Joan Chen, Jamie Ryding, Sherman Chang, Mervyn Bibb

**Affiliations:** 1 Department of Molecular Microbiology, John Innes Centre, Norwich Research Park, Colney, Norwich, NR4 7UH, UK; 2 Verenium Corporation, San Diego, CA 92121, USA; 3 Biology Department, San Diego State University, San Diego, CA 92182, USA; 4 Dermtech International, San Diego, CA 92121, USA

## Abstract

**Background:**

Regulation of production of the translational apparatus via the stringent factor ppGpp in response to amino acid starvation is conserved in many bacteria. However, in addition to this core function, it is clear that ppGpp also exhibits genus-specific regulatory effects. In this study we used Affymetrix GeneChips to more fully characterize the regulatory influence of ppGpp synthesis on the biology of *Streptomyces coelicolor *A3(2), with emphasis on the control of antibiotic biosynthesis and morphological differentiation.

**Results:**

Induction of ppGpp synthesis repressed transcription of the major sigma factor *hrdB*, genes with functions associated with active growth, and six of the thirteen conservons present in the *S. coelicolor *genome. Genes induced following ppGpp synthesis included the alternative sigma factor *SCO4005*, many for production of the antibiotics CDA and actinorhodin, the regulatory genes *SCO4198 *and *SCO4336*, and two alternative ribosomal proteins. Induction of the CDA and actinorhodin clusters was accompanied by an increase in transcription of the pathway regulators *cdaR *and *act*II-ORF4, respectively. Comparison of transcriptome profiles of a *relA *null strain, M570, incapable of ppGpp synthesis with its parent M600 suggested the occurrence of metabolic stress in the mutant. The failure of M570 to sporulate was associated with a stalling between production of the surfactant peptide SapB, and of the hydrophobins: it overproduced SapB but failed to express the chaplin and rodlin genes.

**Conclusion:**

In *S. coelicolor*, ppGpp synthesis influences the expression of several genomic elements that are particularly characteristic of streptomycete biology, notably antibiotic gene clusters, conservons, and morphogenetic proteins.

## Abstract 2

The induction of ppGpp synthesis in Streptomyces coelicolor influenced the expression of several genomic elements characteristic of streptomycete biology, including antibiotic gene clusters, conservons, and morphogenetic proteins.

## Background

Free-living bacteria are at the mercy of environmental conditions, and must possess mechanisms for rapidly responding and adapting to changing circumstances to survive. Streptomycetes are non-motile, mycelial soil bacteria that are unrivalled producers of bioactive secondary metabolites, including a wide variety of antibiotics with important uses in medicine and agriculture. On encountering conditions of famine and unable to actively seek out new sources of nutrients, *Streptomyces *colonies initiate a developmental program that culminates in the production of spores for dispersal, and involves transitioning from vegetative growth on and within the (now exhausted) food substrate to the erection of aerial hyphae (reviewed in [1,2]). Concomitantly, the colonies start producing antibiotics, perhaps to protect for their own use nutrients released upon lysis of a proportion of the substrate hyphae, an event that occurs at the onset of aerial mycelium formation. The regulation of antibiotic production is complex, involving many different families of regulatory proteins, and both extracellular and intracellular signaling molecules (reviewed in [3]).

One important system for sensing nutrient starvation and triggering adaptive responses in bacteria involves the highly phosphorylated guanine nucleotide ppGpp, also known as stringent factor. This has long been known to effect a rapid response to amino acid starvation in *Escherichia coli*, down-regulating both rRNA biosynthesis and ribosome production [4,5]. Under amino acid limiting conditions, the RelA protein associated with ribosomes synthesises ppGpp in response to occupancy of the ribosomal A-site by uncharged tRNAs. The mode of action of ppGpp has been studied extensively in *E. coli*, and involves reorienting gene transcription via binding to RNA polymerase (reviewed in [6]).

In *Streptomyces coelicolor *A3(2), RelA appears to be the only source of ppGpp synthesis [7,8]. Moreover, when grown under nitrogen-limiting conditions, a Δ*relA *mutant is defective in the production of two antibiotics: the blue-pigmented polyketide actinorhodin (Act) and the red pigmented tri-pyrolle undecylprodigiosin (Red); the mutant is also delayed in the onset and extent of morphological differentiation [7]. Hesketh *et al*. [9] used a carboxy-terminally truncated derivative of *relA *expressed from a thiostrepton-inducible promoter to achieve controllable levels of ppGpp production in *S. coelicolor *independently of amino acid starvation, and demonstrated a link between induction of ppGpp synthesis and increased transcription of the activator gene controlling Act biosynthesis, *act*II-ORF4. This supported previous work in a number of different *Streptomyces *species where ppGpp had been shown to influence antibiotic biosynthesis [10-14]. The suggestion that ppGpp serves to regulate cellular functions other than ribosome biogenesis agrees with the results of studies in other bacterial species, where it plays a role in diverse processes, including social behavior (quorum sensing and biofilm formation), pathogenesis, symbiosis, stress survival and morphological development (reviewed in [15]). Indeed, in *E. coli*, ppGpp is now considered much more as a global regulator rather than simply as a regulator of ribosome production, redirecting transcription so that genes important for starvation survival and virulence are favored at the expense of those required for growth and proliferation [6].

The purpose of this study was to use methods for the genome-wide analysis of gene transcription to more fully characterize the regulatory influence of ppGpp synthesis on the biology of *S. coelicolor*, with particular emphasis on the processes of morphological differentiation and secondary metabolite production. Classically, the effects of ppGpp have been analysed following induction of ppGpp production via starvation for one or more amino acids. This complicates interpretation of the results since the changes observed include responses both to the increase in ppGpp concentration, and to the ppGpp-independent effects of starvation. The levels of ppGpp produced in this way are also often artificially high in comparison to those observed when starvation occurs naturally. In this work we utilize a system that enables controlled induction of more physiologically relevant levels of ppGpp in the absence of amino acid starvation, allowing the effects of ppGpp synthesis to be viewed in isolation. This is supplemented by a comparison of *relA*+ (ppGpp+) and *relA*- (ppGpp-) strains to observe the longer term differences in gene expression resulting from an absence of ppGpp synthesis, and how this affects the transition to antibiotic production and morphological differentiation during growth. The results extend the known involvement of ppGpp synthesis in the regulation of antibiotic and secondary metabolite production, and paint a picture of a global regulatory mechanism with inhibitory and stimulatory effects on the transcription of a broad range of genes with diverse cellular functions. Although the direct regulatory routes remain unclear, it appears that, at least under certain growth conditions, ppGpp synthesis is required for correctly redirecting and coordinating gene transcription in *S. coelicolor *to allow it to progress normally through its developmental life-cycle.

## Results and discussion

### Description and overview of datasets

To determine the effect of ppGpp synthesis on global gene expression in *S. coelicolor *we used two complementary strategies. In the first approach, to study the immediate effects of ppGpp production, we activated ppGpp synthesis in exponentially growing cells in the absence of amino acid starvation by using a strain (M653 [Δ*relA tipAp::relA*(1.46 kb)]) that expresses a truncated portion of *relA *under the control of a thiostrepton-inducible promoter [9]. Samples were harvested at 30 minute intervals following induction for comparison to a control set of samples from aliquots of the same cultures that were not induced. Dry cell weight measurements in the two sets of cultures were similar, indicating that the induction of ppGpp synthesis had no gross effect on growth. As a control for the effect of thiostrepton on gene expression, a similar study was undertaken using strain M667 [Δ*relA tipAp::*], which lacks the truncated *relA *gene downstream of the thiostrepton-inducible promoter but grows at a similar rate to strain M653 [Δ*relA tipAp::relA*(1.46 kb)] [9]. In the second approach, we sampled cultures of a *relA *deletion mutant strain that is completely defective in the ability to synthesise ppGpp, but which shows no growth rate defect [7], during growth on a complex medium over a five day period. These were compared to similar samples of the parent strain grown under the same conditions. Details of the microarray data analysis methods are given in the Materials and methods.

#### Changes in gene expression upon induction of ppGpp synthesis in M653 [Δ*relA tipAp::relA*(1.46 kb)]

**Figure 1:**
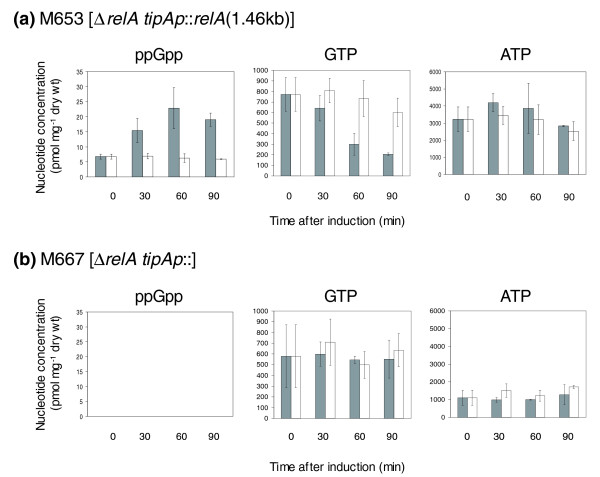
Changes in intracellular nucleotide concentrations in induced (grey bars) and non-induced (black bars) cultures of **(a) **M653 [Δ*relA tipAp::relA*(1.46 kb)] and **(b) **M653 [*ΔrelA tipAp::*]. Cultures were grown to an OD_450 _of approximately 0.5 before treatment with 25 μg ml^-1 ^thiostrepton (induced) or DMSO (non-induced), and intracellular levels of nucleotides measured by HPLC analysis of extracts of cells harvested at 0, 30, 60 and 90 minutes. Values shown are in pmol mg^-1 ^dry cell weight and are the average of biological triplicate experiments for (a) and duplicates for (b), with standard deviations marked with error bars.

Induction of exponentially growing cultures of *S. coelicolor *strain M653 [Δ*relA tipAp::relA*(1.46 kb)] by treatment with thiostrepton (25 μg ml^-1^) resulted in an approximately three-fold increase in intracellular ppGpp concentration after 60-90 minutes (Figure 1a). The maximum concentration achieved was approximately 25 pmol mg^-1 ^dry cell weight, which is 15-20% of the levels typically obtained following starvation of actively growing *S. coelicolor *by amino acid shift-down but similar to those measured in cultures naturally progressing to starvation during transition to stationary phase [16]. Control cultures to which thiostrepton was not added were consistently low in ppGpp over the same period, at around 6 pmol mg^-1 ^dry weight at all times, attributable to synthesis derived from basal expression of the *tipA *promoter. This is approximately three- to six-fold higher than the amount of ppGpp usually detected in the wild-type strain under similar conditions, but has no observable effect on growth rate [9]. Levels of GTP in the induced cultures showed a three-fold decrease over the 90 minute period studied, but remained approximately constant in the non-induced cells. The fall in GTP upon stimulation of ppGpp synthesis in *S. coelicolor *is consistent with previous results (for example, [7]), and is at least in part due to conversion of GTP to ppGpp. Since it is not possible to elicit ppGpp synthesis without also causing a reduction in GTP concentrations, downstream effects of ppGpp synthesis on gene expression reported in this work could in principle be attributable to the change in concentration of either nucleotide. ATP concentrations were similar between the two experiments, and also did not change significantly with time. In contrast, in the control strain M667 [Δ*relA tipAp::*] ppGpp was not detected in any sample, and levels of GTP were similar in the induced and non-induced cultures (Figure 1b). ATP concentrations were again similar in these two sets of cultures, and also did not change significantly with time, but were generally lower in M667 [Δ*relA tipAp::*] than in M653 [Δ*relA tipAp::relA*(1.46 kb)].

RNA was extracted from the same cultures used for the nucleotide analysis detailed above, and gene expression measurements obtained by hybridization to Affymetrix diS_div712a GeneChips containing oligo probes for 97% of the 7,825 protein-encoding genes in *S. coelicolor*. Data analysis revealed a total of 752 genes whose expression profiles were significantly influenced by the induction (Additional data file 1). Genes in this list include not only those affected as a result of induction of ppGpp synthesis, but also those changed in abundance as a result of thiostrepton addition. Using strain M667 [Δ*relA tipAp*::] it was possible to identify those genes altered by the addition only of thiostrepton (see Materials and methods), resulting in a final list of 589 genes that had been significantly affected by induction of ppGpp synthesis alone. To reduce the number of genes for consideration and to focus in on only the largest changes, the data for these 589 genes were subjected to further tests (see Materials and methods). These were based on analysing fold-change ratios between induced and non-induced samples to identify those that are clearly induced or repressed by ppGpp synthesis, and gave lists of 98 and 189 genes, respectively (Additional data file 2). These lists of genes were analysed further to identify over-represented (*P *< 0.05) pathways or functions (Tables S1 and S2 in Additional data file 3).

#### Changes in gene expression between non-induced samples of strains M653 and M667

Non-induced cultures of strain M653 [Δ*relA tipAp::relA*(1.46 kb)] exhibit a constitutively low level of ppGpp synthesis (about 6 pmol mg^-1 ^dry weight) whereas those of the control strain M667 [Δ*relA tipAp::*] are completely defective in ppGpp production (Figure 1). The two strains grow at a similar rate [9]. Comparison of the datasets for these non-induced samples should, therefore, reveal changes in global gene expression resulting from intracellular ppGpp levels changing from 0 to 6 pmol mg^-1 ^dry weight, supplementing the results from the induction experiments, which involved increases in ppGpp concentrations from approximately 6 to 25 pmol mg^-1 ^dry weight. Data analysis identified 428 genes that were significantly (*P *< 0.01) differentially expressed between the two strains (Additional data file 4). Of these genes, 76 were selected by visual inspection as clearly more highly expressed in strain M653 (ppGpp = 6 pmol mg^-1 ^dry weight) compared to M667 (0 pmol mg^-1 ^dry weight), while 352 genes were expressed at lower levels. Both lists of genes were analysed further to identify over-represented (*P *< 0.05) pathways or functions (Tables S3 and S4 in Additional data file 3).

#### Changes in gene expression as a result of the *relA *mutation

**Figure 2:**
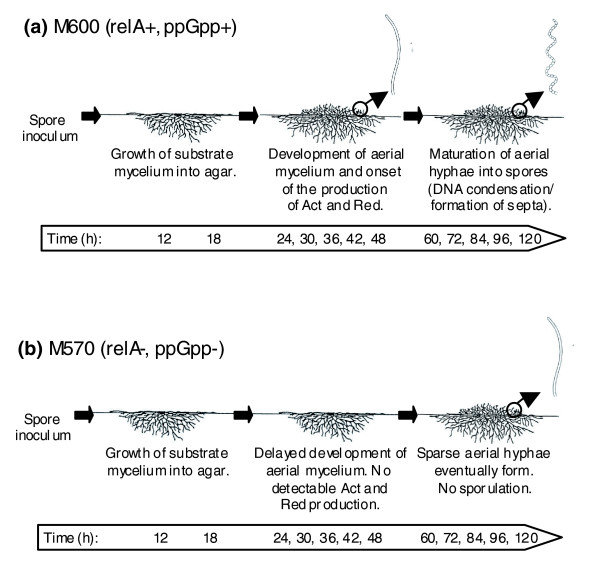
Illustration of the growth and sampling of cultures of **(a) **M600 (*relA*+ ppGpp+) and **(b) **M570 (*relA*- ppGpp-) on MYM TE agar. M600 progressed normally through its developmental cycle, beginning to erect aerial hyphae after 24 h and to produce the antibiotics Red after 36 h and Act after 48 h. Grey spores were also detectable from 60 h onwards. M570 failed to produce detectable amounts of pigment, and formed only a very sparse covering of aerial mycelium, first observable at 24 h. Samples 1-8 correspond to 12, 24, 36, 48, 60, 84, 96, and 120 h, respectively.

Gene expression patterns during growth on a rich nutrient agar medium (MYMTE) were compared between M570, a *relA *deletion mutant strain that is completely defective in the ability to synthesise ppGpp [7,8], and the parental strain M600. MYMTE was selected since the mutant strain is clearly defective in both morphological differentiation and production of pigmented antibiotics when cultured on this medium (Figure 2). M600 progressed normally through its developmental cycle, beginning to erect aerial hyphae after 24 h, and to produce Red after 36 h and Act after 48 h. Grey spores were detectable by microscopy from 60 h onwards. M570 failed to produce detectable amounts of pigment at any time, and formed only a very sparse covering of aerial mycelium, observable after 24 h. It did not sporulate in the duration of the experiment, although when grown on MYMTE lacking cellophane discs it exhibited a significant delay in sporulation rather than a complete deficiency. RNA samples were isolated from cultures of each strain at 12 time points during growth, and gene expression profiles compared following hybridization to microarrays. Quality control of the array data failed two chips, corresponding to replicate 2 of the 60 h sample for both M600 and M570, and these were therefore omitted from further analysis. A further 12 chips, for M600 cultures harvested after 18, 30, 42, and 72 h, were processed separately from the other 60 samples, and when the results were displayed in GeneSpring they exhibited subtly different expression levels for a minor subset of genes when compared to the other M600 samples. These, and the M570 data from the corresponding times, were therefore omitted from the detailed statistical consideration of the data, although they were used as a resource to supplement information on gene expression trends when necessary.

Two-way ANOVA analysis of the filtered data from the 12, 24, 36, 48, 60, 84, 96, and 120 h samples identified 2,031 genes that were significantly differentially expressed at the 1% probability level according to strain only, 3,074 genes according to time only and 1,033 genes according to a combination of strain and time (Additional data file 5). The 2,031 genes significantly altered by mutation in *relA *represent approximately 25% of the genome and indicate extensive alterations in patterns of gene expression in the mutant strain. Cluster analysis can be used to identify groups of genes that are either co-ordinately controlled or participate in common cellular processes. These genes were therefore clustered according to their expression profiles using the QT (quality threshold) clustering algorithm, applying a requirement for a minimum Pearson correlation of 0.9 and minimum cluster size of 5 genes. This produced 100 clusters containing a total of 1,093 genes, with 92 genes present in the largest cluster (Additional data file 6). The upstream regions of genes in each QT cluster were analysed for common promoter elements as detailed in the Materials and methods and those referred to in the text are noted in Additional data file 6.

The list of significantly differently expressed genes was further analysed as detailed in the Materials and methods to identify biological pathways significantly over-represented (*P *< 0.05) by the data (Table S5 in Additional data file 3).

### ppGpp synthesis represses many genes associated with active growth, transport processes, and conservons in *S. coelicolor*

**Figure 3:**
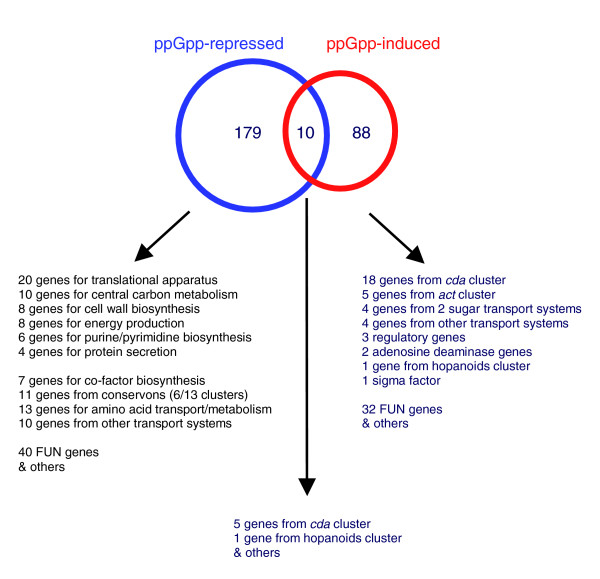
Induction of ppGpp synthesis in *S. coelicolor *represses genes associated with active growth, but stimulates transcription from the *act *and *cda *antibiotic clusters.

Classically, the stringent response mediated by ppGpp involves a reduction in rRNA biosynthesis and ribosome production, and stringent control of the *rrnD *rRNA gene set in *S. coelicolor *has been reported [16]. Probes for rRNA operons are not present on the microarrays used, but the identification of 14 genes encoding ribosomal proteins, plus 6 also associated with ribosome biogenesis and function, in the list of 189 ppGpp-repressed genes following induction in strain M653 [Δ*relA tipAp::relA*(1.46 kb)] is also consistent with this occurring in *S. coelicolor *(Figure 3, Additional data file 2). Indeed, ribosome production was top of the list of pathways and processes repressed by ppGpp induction (Table S1 in Additional data file 3). Moreover, the data indicate that a further 51 genes whose functions are clearly associated with active cell growth, that is, carbon metabolism, oxidative phosphorylation, cell wall biosynthesis, ATP synthesis, fatty acid biosynthesis, purine/pyrimidine biosynthesis, co-factor production and amino acid biosynthesis were also repressed. This suggests an extended role for ppGpp in *S. coelicolor *in coordinating the suppression of processes associated with cell proliferation, even in the presence of sufficient nutrients to support exponential growth. A global proteome/transcriptome analysis of the response of *Bacillus subtilis *to ppGpp synthesis induced in exponentially growing cells by addition of the leucyl- and isoleucyl-tRNA aminoacylation inhibitor DL-norvaline reported similar results [17]. However, the observed repression of genes involved in central carbon metabolism and purine/pyrimidine biosynthesis in *B. subtilis *was said to occur independently of *relA*, and, therefore, presumably also of ppGpp synthesis, in contrast to our findings in *S. coelicolor*. In *Corynebacterium glutamicum*, transcription of the majority (though not all) of the ribosomal protein genes is reported to be controlled in a *rel*-independent manner, and the list of stringently controlled genes is instead dominated by those with a role in nitrogen metabolism [18]. This suggests some degree of variation between genera in the core functions regulated by ppGpp. Conway and co-workers have also reported a central role for ppGpp in coordinating the global down-regulation of sets of genes involved in active growth in *E. coli *during glucose-lactose diauxie, and in response to growth arrest induced by H_2_O_2_, and proposed a model wherein the ppGpp-dependent redistribution of RNA polymerase across the genome is the driving force behind control not only of the stringent response, but also the general stress response and starvation-induced carbon scavenging [19,20]. In this study, the observed ppGpp-dependent down-regulation of the Sec protein secretion apparatus, plus 16 other genes encoding proteins with transport functions, also suggests a significant role for ppGpp in *S. coelicolor *in reprogramming the import/export of nutrients. An additional eight genes encoding putative transporters were also found to be induced by ppGpp synthesis (see below). Interestingly, the ROK-family transcriptional repressor SCO6008 was ppGpp-repressed while the first gene from the adjacent putative carbohydrate transport operon SCO6005-6007 [21] was ppGpp-induced, perhaps suggesting that expression of this operon is usually repressed by SCO6008. A two-fold repression of SCO6008 following induction of ppGpp synthesis in M653 [Δ*relA tipAp::relA*(1.46 kb)] was confirmed by quantitative RT-PCR (qRT-PCR; data not shown).

Biosynthesis of the vitamin B12 co-factor appears to be at least partially regulated by ppGpp in *S. coelicolor *(Additional data file 2 and Table S1 in Additional data file 3), with three genes from the *cob *locus at SCO1847-1859 being ppGpp-repressed. Although not present in the significantly differently expressed genes in the array data, qRT-PCR confirmed that the first gene in the putative SCO1847-53 transcription unit that comprises half of this locus was approximately 2-fold reduced 60 minutes after induction of ppGpp synthesis in M653 [Δ*relA tipAp::relA*(1.46 kb)] (data not shown). In addition, many genes in this cluster were significantly down-regulated in strain M600 compared to the *relA *mutant strain (QT52 in Additional data file 6), and 6 of the 38 genes identified as possessing B12 riboswitches [22] were repressed upon induction of ppGpp synthesis.

Interestingly, 11 genes associated with 6 of the 13 conservons (cvns) present in the genome of *S. coelicolor *were ppGpp-repressed. Cvns, first identified in *S. coelicolor *by Bentley *et al*. [23], are conserved operons typically consisting of four genes, two of unknown function sandwiched between a sensor histidine kinase homologue and a gene encoding an ATP/GTP-binding protein. They are also present in the genomes of *Streptomyces avermitilis *(12 copies) [24] and *Streptomyces scabies *(13 copies) [25], in some cases with cytochrome P450 genes associated with them, and to date have only been found in the genomes of *Actinomycetales *[26]. Genes from cvn1, cvn4, cvn6, cvn10 (the cytochrome genes only), cvn12 and cvn13 were repressed following ppGpp synthesis, while none were identified in the ppGpp-induced list. qRT-PCR analysis of the samples taken 60 minutes following induction confirmed that transcription of the first genes from each of cvns 1, 10 and 13 were reproducibly approximately two-fold or more repressed following induction of ppGpp synthesis in strain M653 [Δ*relA tipAp::relA*(1.46 kb)] (Table 1; Figure S1 in Additional data file 7). Mutation of the ATP/GTP-binding homologue in cvn9 of *S. coelicolor *affected both morphological differentiation and production of pigmented antibiotics, as did mutation of the kinase homologue of cvn9 or cvn10 [26,27]. The suggested signaling role for cvns is supported from the results of a biochemical analysis that indicates that the proteins from cvn9 comprise a membrane-associated hetero-complex resembling the eukaryotic G-protein-coupled receptor system [26]. Twenty-one genes from a total of eight cvns, including all genes from cvn9, were significantly altered in their transcription when comparing the parent (ppGpp+) and *relA *mutant (ppGpp-) strains (Table S5 in Additional data file 3, and Additional data file 5), and it is interesting to speculate that some of the wide-ranging effects on transcription that are exerted by ppGpp may be mediated via controlling the level of expression of the cvns. Given the reported influence of certain cvns over production of the pigmented antibiotics in *S. coelicolor*, it is also possible that ppGpp exerts at least some of its effects on the regulation of Act and Red synthesis via this route. The ATP/GTP-binding protein homologue present as the fourth gene in each cvn has both GTP-hydrolysing and GTP/GDP-binding activities [26], and the decrease in GTP concentration associated with the synthesis of ppGpp could also influence any signaling activity of the cvns.

**Table 1:** qRT-PCR analysis of the transcription of cvns 1, 10 and 13

		M653 transcript abundance ratio 0/I60*		M667 transcript abundance ratio 0/I60*	
			
Gene	Cvn number	Induction replicate R1	Induction replicate R2	Induction replicate R1	Induction replicate R2
*SCO5544*	1	1.78	1.95	0.97	0.66
*SCO7422*	10	5.26	1.70	0.98	0.72
*SCO7463*	13	5.26	2.87	0.92	0.71

*The value for transcript abundance (measured by qRT-PCR) immediately prior to induction (0 minutes) divided by the abundance 60 minutes after addition of thiostrepton to the culture (I60). The data confirm that transcription of the first gene in each of cvns 1, 10 and 13 is repressed following induction of ppGpp synthesis in M653 [Δ*relA tipAp::relA*(1.46 kb)] but unaffected in the control strain M667 [Δ*relA tipAp::*].

Eight genes whose annotated function is associated with amino acid biosynthesis were significantly repressed following induction of ppGpp synthesis in M653 [Δ*relA tipAp::relA*(1.46 kb)] (Additional data file 2). These are *hisC1*, *aroB*, *dapB*, *thrB*, *argH*, *glyA1*, *cysD and cysH*. Previous reports in different organisms have also indicated a role for ppGpp in the regulation of at least some amino acid biosynthesis genes, although both positive and negative effects have been reported depending on the organism and the amino acid. In *C. glutamicum*, both the histidine and serine biosynthetic genes are under strong positive stringent control [18], and the *his *operon in *E. coli *and *Salmonella typhimurium *is de-repressed following accumulation of ppGpp [28,29]. However, stringent control of serine and histidine biosynthetic gene expression was not observed in *B. subtilis*, but genes associated with the biosynthesis of branched chain amino acids did exhibit a RelA-dependent induction [17]. In contrast, glutamine synthetase I is negatively stringently controlled in *C. glutamicum *[18], and in *E. coli *approximately one-half of the genes encoding amino acid biosynthetic enzymes are down-regulated in response to growth arrest [19].

### Transcription of the major vegetative sigma factor σ-*hrdB *is repressed following ppGpp synthesis, while the alternative ECF sigma factor σ-*SCO4005 *is induced

**Figure 4:**
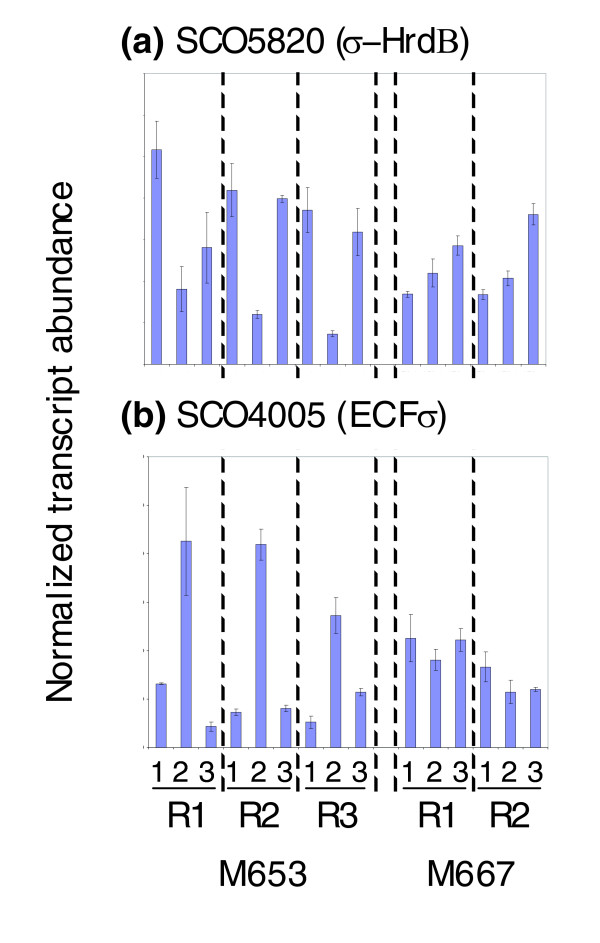
qRT-PCR shows transcription of the major vegetative sigma factor *hrdB *is repressed following induction of ppGpp synthesis, while the alternative ECF sigma factor encoded by *SCO4005 *is induced. In each biological replicate experiment, R1-R3, using strain M653 [Δ*relA tipAp::relA*(1.46 kb)] or the control strain M667 [Δ*relA tipAp*], lane 1 corresponds to the pre-induction sample (0 minutes) and lanes 2 and 3 correspond to the samples taken 60 minutes after induction or non-induction with thiostrepton, respectively. The average of three qRT-PCR determinations is shown, and standard deviations are marked with error bars.

Sigma factors dictate selection of gene transcription by RNA polymerase by specifying recognition of only certain promoter sequences. σ-HrdB is essential for cell viability, and is the major sigma factor for transcription of genes required for active, vegetative growth in *S. coelicolor *[30]. Although not represented on the GeneChip used in the microarray analyses, transcription of *σ-hrdB *was analysed using qRT-PCR and found to be approximately three- to four-fold repressed 60 minutes after induction of ppGpp synthesis in M653 [Δ*relA tipAp::relA*(1.46 kb)] (Figure 4a). It was not significantly affected in the control experiments. In similar studies looking at stringent control of gene expression in *E. coli *[19,20], *B. subtilis *[17] and *C. glutamicum *[18], transcription of the principal vegetative sigma factors was not found to be significantly stringently controlled. The alternative extra-cytoplasmic function (ECF) sigma factor *σ-SCO4005 *is among the 98 genes identified as being significantly induced following ppGpp synthesis in *S. coelicolor *(see below); this was confirmed by qRT-PCR, which indicated a three- to five-fold increase in transcription 60 minutes after the induction of ppGpp production (Figure 4b). Two alternative sigma factors have previously been reported as being positively stringently controlled in other bacteria: the stationary phase sigma factors RpoS in *E. coli *[31,32] and SigB in *C. glutamicum *[18]. The stationary phase sigma factor in *B. subtilis *is not directly regulated by ppGpp [33], although there is evidence that its activity can be regulated in a RelA-dependent manner [34]. In *S. coelicolor*, the four-fold decrease in *σ-hrdB *transcription following ppGpp synthesis has the potential to strongly influence the promoters selected for transcription by RNA polymerase, thereby leading to significant re-orientation of genome expression. The concomitant and corresponding increase in expression of *σ-SCO4005 *can readily be imagined to further contribute to this, although the extent of this contribution is currently unknown. It is clear however that *SCO4005 *is not involved in mediating the increase in expression of the Act cluster that follows induction of ppGpp synthesis, since induction of ppGpp in a *relA SCO4005 *double mutant strain results in an increase rather than a decrease in Act production (data not shown).

### ppGpp synthesis induces transcription of the *act *and *cda *antibiotic biosynthesis clusters, the hopanoids cluster and a limited number of genes with regulatory functions

In contrast to ppGpp-repression, the list of genes induced following ppGpp synthesis is dominated by those associated with secondary metabolic processes (Figure 3; Table S2 in Additional data file 3). Of the 98 identified as ppGpp-induced in strain M653 [Δ*relA tipAp::relA*(1.46 kb)], 23 belong to the cluster of genes responsible for producing the antibiotic CDA, 5 are from the Act antibiotic biosynthetic cluster, and 2 are from the hopanoids cluster. This is the first report linking ppGpp synthesis to the regulation of the *cda *cluster, while ppGpp-dependent induction of the *act *cluster has previously been documented, acting via increasing transcription of the pathway regulator *act*II-ORF4 [9]. No effect on transcription of the Red biosynthetic gene cluster was observed. Although not in the list of significantly ppGpp-induced genes, the array data show an upward trend for transcription of the pathway-specific regulatory gene controlling CDA production, *cdaR*, following the initiation of ppGpp synthesis, and qRT-PCR confirmed that it was induced two- to four-fold in a ppGpp-dependent manner, similar to *act*II-ORF4 (Figure S2 in Additional data file 7). qRT-PCR also verified the induction of the CDA non-ribosomal peptide synthase I gene, *SCO3230*, following ppGpp synthesis (data not shown). Four other genes with putative regulatory functions (*SCO4005*, *SCO4198*, *SCO4263 *and *SCO4336*) were also significantly induced by ppGpp, and it is formally possible that they play a role in mediating the ppGpp-dependent rise in transcription of the *act*II-ORF4 and *cdaR *regulators. The induction in transcription of *SCO4005*, *SCO4198*, and *SCO4336 *was confirmed by qRT-PCR (Figure 4; Figure S3 in Additional data file 7). Transcription of the sigma factor gene *SCO4005 *is, however, paradoxically significantly up-regulated in the ppGpp- deficient strain M570 (see below), and insertion mutagenesis of *SCO4005 *produced no change in Act production (data not shown). Similar mutant strains carrying transposon insertions in the DNA-binding protein gene *SCO4198 *or the MarR-family regulatory gene *SCO4336 *were reduced in their ability to synthesise Act, but only on certain media (data not shown). A deletion mutant of *SCO4263*, a TTA-containing regulatory gene, possesses no antibiotic production phenotype [35]. Transcript abundances of regulatory genes previously reported to positively influence expression of *act*II-ORF*4 *and/or *cdaR*, including *afsR *[36,37], *afsS *[38,39], *scbR *[40], and *SCO4118 *[41], were not significantly altered by induction of ppGpp synthesis. In particular, qRT-PCR and S1 nuclease protection analysis confirmed that transcription of *SCO4118*, encoding a TetR-family regulator known to bind to the promoter of *act*II-ORF4 and activate its transcription [41], was unaffected at levels of ppGpp induction resulting in significant increases in transcription of the *act *cluster (data not shown). It therefore appears that ppGpp is not acting on the CDA and Act clusters via transcriptional control of these regulators (although post-transcriptional effects cannot be ruled out), and a direct effect on pathway-regulator promoter activity seems more likely. However, it is interesting to note that transcription of *SCO6264*, a reductase immediately adjacent to the *scbR*-*scbA *locus, is up-regulated following ppGpp synthesis, with a two-fold or higher induction confirmed by qRT-PCR (Figure S4 in Additional data file 7). The enzyme encoded by this gene is believed to play a role in modification of the γ-butyrolactone signaling molecule putatively synthesised by ScbA and known to influence production of both Act and Red [40,42]. A *SCO6264 *deletion mutant is defective in the synthesis of γ-butyrolactones (T Nihara, personal communication).

Production of hopanoids in *S. coelicolor *occurs during the transition from substrate to aerial hyphae, and they have been proposed to play a role in alleviating stress associated with membrane permeability [43]. The observed activation of the hopanoid biosynthetic cluster upon ppGpp synthesis could similarly represent a response to physiological stress.

Ten genes were found in both the ppGpp-repressed and the ppGpp-induced gene lists, including five from the *cda *cluster. All appear repressed in the 30 minute sample, but induced in the 60 and 90 minute samples, possibly reflecting different responses to the intracellular concentration of ppGpp, which after 30 minutes is intermediate between the pre-induction level and the maximum achieved in the later two time points.

### ppGpp synthesis induces transcription of two genes encoding alternative ribosomal proteins with a putative role in zinc homeostasis

**Figure 5:**
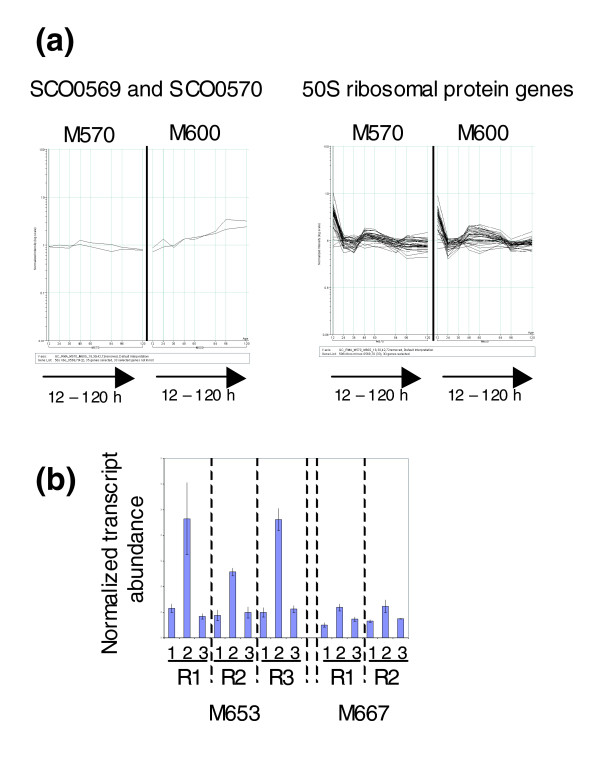
ppGpp synthesis and the expression of alternative ribosomal protein genes.**(a) **Different growth-phase dependent expression of genes encoding the alternative ribosomal proteins *SCO0569 *and *SCO0570 *compared to the 50S ribosomal protein genes. In each panel, the x-axis represents culture age, and the y-axis is normalized transcript abundance in log_10 _scale. **(b) **qRT-PCR shows transcription of *SCO0570 *is activated following induction of ppGpp synthesis. In each biological replicate experiment, R1-R3, using strain M653 [Δ*relA tipAp::relA*(1.46 kb)] or the control strain M667 [Δ*relA tipAp*], lane 1 corresponds to the pre-induction sample (0 minutes), and lanes 2 and 3 correspond to the samples taken 60 minutes after induction or non-induction with thiostrepton, respectively. The average of three qRT-PCR determinations is shown, and standard deviations are marked with error bars.

In contrast to the general trend for transcription of genes associated with ribosome biogenesis to be repressed by ppGpp, the ribosomal protein gene *SCO0569 *was induced by the stringent factor following induction of the *tipAp::relA *construct in strain M653 [Δ*relA tipAp::relA*(1.46 kb)]. *SCO0569 *(*rpmJ2*) is predicted to encode an alternative form of the L36 ribosomal protein specified by *SCO4726 *(*rpmJ1*). The major difference between the two forms is that *SCO0569 *lacks cysteine residues and does not contain the CxxC zinc-binding motif present in *SCO4726 *[44]. Transcription of *SCO0569 *was also significantly different in the growth curve comparison of M600 (*relA*+) and M570 (*relA-*), exhibiting a lower level of expression in the mutant, and the pattern of expression in the parent strain was different to the majority of ribosomal protein genes (Figure 5a). The adjacent ribosomal protein gene *SCO0570 *(*rpmG3*) encodes an analogous cysteine-less alternative to the RpmG protein, and has a similar pattern of expression to *SCO0569 *in the parent strain. Although not present in the initial list of genes induced by ppGpp, qRT-PCR indicates that it is in fact positively stringently controlled, showing an approximately four-fold increase in transcription 60 minutes after induction (Figure 5b).

In *B. subtilis*, the ability to replace certain ribosomal proteins possessing zinc-binding motifs with alternative versions lacking this property has been proposed to play a role in zinc homeostasis, causing the release of the metal ions locked up in the ribosome when conditions are limiting [45,46]. The existence of alternative ribosomal proteins that do not require zinc for their function is also thought to provide a fail-safe mechanism for *de novo *synthesis of ribosomes under zinc-limiting conditions [47]. Recent work in *S. coelicolor *where a zinc-specific regulator, Zur, was shown to control the expression of at least five such alternative ribosomal proteins suggests that a similar system operates in streptomycetes [48,49]. Our results indicate that ppGpp may have a role to play in these processes in *S. coelicolor*, promoting the synthesis of non-zinc-dependent ribosomes and increasing intracellular zinc concentrations during times of nutritional stress through induction of *SCO0569 *(*rpmJ2*) and *SCO0570 *(*rpmG3*) transcription. Owen *et al. *[48] found that *SCO0569 *and *SCO0570 *are co-transcribed from a single promoter that is controlled by the alternative sigma factor SigR rather than Zur, and it is possible that ppGpp mediates its effect on transcription of these genes via SigR. However, transcription of *sigR *is unaffected following induction of ppGpp synthesis, suggesting the influence is post-transcriptional, or mediated via an as yet unidentified SigR-independent promoter.

### The phenotypic differences between M600 (*relA*+ ppGpp+) and M570 (*relA*- ppGpp-) during growth on MYMTE are reflected in the significantly differently expressed genes identified in the transcriptome data

#### Genes associated with morphological differentiation

**Figure 6:**
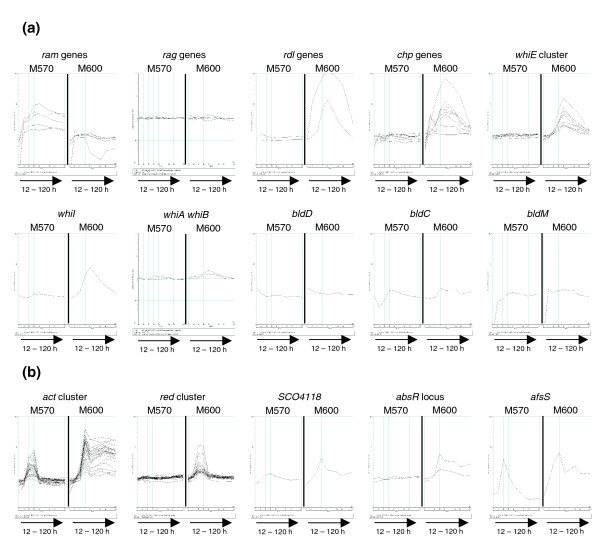
Transcription profiles of genes associated with **(a) **morphological differentiation, and **(b) **pigmented antibiotic production that were significantly differently expressed between strain M600 (*relA*+ ppGpp+) and M570 (*relA*- ppGpp-). In each panel, the x-axis represents culture age, and the y-axis is normalized transcript abundance in log_10 _scale.

**Figure 7:**
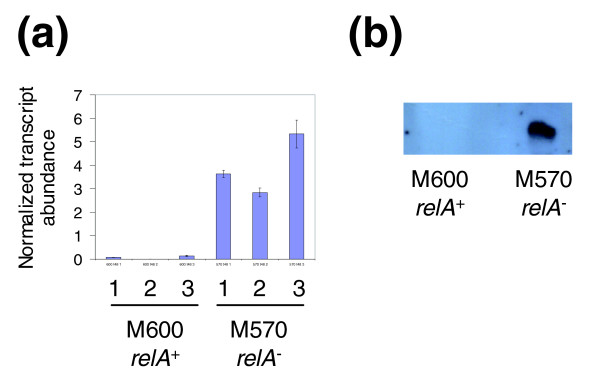
Comparison of SapB expression in M600 (*relA*+ ppGpp+) and M570 (*relA*- ppGpp-). **(a) **qRT-PCR confirms that transcription of *sapB *in the 48 h samples is approximately 40-fold increased as a result of *relA *mutation. **(b) **This is reflected in a large increase in SapB extracted from M570 (*relA*-) cells harvested after 48 h growth on MYM TE agar, as detected by Western blotting. Lanes 1, 2 and 3 indicate biological replicate samples. The average of three qRT-PCR determinations is shown, and standard deviations are marked with error bars. Western analysis was performed on biological duplicate samples, and a representative result is shown.

Mutants of *S. coelicolor *that lack an obvious aerial mycelium are called *bld *(for bald), while those that produce an aerial mycelium but do not generate normal mature spores are called *whi *(for white, reflecting a lack of grey spore pigment). Studies of *bld*, *whi *and other mutant strains have established models for the regulation of morphological development in *S. coelicolor *(reviewed in [1,2]), where the *bld *cascade controls checkpoints that eventually lead to the onset of aerial growth, resulting in the formation of surface active molecules that lower the water surface tension enabling hyphae to break free and grow into the air. Once aerial, the hyphae are then covered with self-assembling layers of hydrophobic proteins (hydrophobins) encoded by the rodlin (*rdl*) and chaplin (*chp*) genes, and subsequently differentiate into chains of unigenomic spores in a process dependent on the *whi *genes. Interestingly, the transcriptome data suggest that M570 (*relA*- ppGpp-) fails to fully erect aerial hyphae and generate spores because it is stalled between the two processes of surfactant synthesis, and coating of the aerial hyphae with hydrophobins (Figure 6a). The *ram *genes (*SCO6681-85*) responsible for the production of the surfactant peptide SapB [50] were significantly over-expressed in M570 from 24 h onwards, whereas transcription of the *rdl *genes and seven of the eight *chp *genes (the exception being *chpB*) was massively reduced in the mutant strain. qRT-PCR analysis of the 48 h culture samples confirmed a reproducible 40-fold or higher over-expression of *sapB *in M570 when compared to the parent strain; a comparable increase in the level of the corresponding protein product present in extracellular extracts at this time was confirmed by Western blotting (Figure 7). This is presumably the result of increased transcription of the regulator of the *ram *cluster, *ramR *(*SCO6685*), observed in strain M570; conceivably, transcription of *ramR *may be directly linked to the nitrogen nutritional status of the cell via ppGpp synthesis. RamR is also known to activate transcription of the *rag *cluster SCO4072-75 that modulates both aerial hyphae formation and sporulation in *S. coelicolor *[51]. Interestingly, however, this operon is not over-expressed in M570 relative to the parent strain, suggesting that an increase in *ramR *transcription alone is not always sufficient for its activation (Figure 6a). Perhaps this division in the two processes regulated by RamR is the root cause of the stalling in the morphological differentiation of M570 when grown on MYMTE. Mutation of *SCO4005 *in the M570 background had no affect on SapB levels (detected by western blotting; data not shown), and SapB overproduction in the *relA *mutant is, therefore, not associated with the observed over-expression of the ECF sigma factor gene.

Transcription of the *whiE *genes specifying production of the grey polyketide spore pigment of *S. coelicolor *was predictably absent in M570, while transcripts of *whiA *(*SCO1950*), together with those of the regulatory genes *whiB *(*SCO3034*) and *whiI *(*SCO6029*), were also significantly reduced. In addition, three of the seven *bld *genes, *bldD*, *bldC *and *bldM*, were significantly differently expressed between the two strains, with the transcriptional repressor *bldD *consistently reduced in its expression in M570 compared to the parental strain from 24 h onwards.

#### Genes associated with production of the pigmented antibiotics

Genes encoding the enzymes, transport systems and pathway-specific regulatory elements necessary for the production of the blue- (actinorhodin) or red- (undecylprodigiosin) pigmented antibiotics are found within the *act *(*SCO5071*-*92*) and *red *(*SCO5877*-*98*) gene clusters, respectively. Previous studies of a *relA *null mutant grown in liquid culture indicated diminished levels of transcription of the regulatory genes *act*II-ORF4 and *redD *[7,9]. In this study, the peak in transcription of genes from the *red *cluster observable in the parent strain at 42 h is completely absent in the mutant (Figure 6b). Transcription of the *act *cluster genes, however, appears to be switched on to almost similar levels in both M600 (*relA+ *ppGpp+) and M570 (*relA*- ppGpp-), but where transcription persists to 120 h in the parent, levels in the mutant decrease to a minimum by 60 h and production of the blue pigment is not observed. Induction of ppGpp synthesis has previously been reported to increase transcription of the *act *cluster regulator *act*II-ORF4 [9], and similar results were also found in this study (see below), offering an explanation for the observed differences in expression of the *act *cluster between the ppGpp+ (M600) and ppGpp- (M570) strains. Genes with a reported role in controlling actinorhodin production, and perhaps also influencing *act*II-ORF4 transcription in this experiment, include *SCO4118 *[41], the *absR *locus (*SCO6992*-*93*) [52], *afsR *[36,37] and *afsS *[38,39]. With the exception of *afsR *(which functions via changes in the phosphorylation state of the gene product: reviewed in Horinouchi [53]), all showed significantly reduced levels of transcription in M570 (Figure 6b). However, knockout mutants in *afsS*, *SCO4118 *and the *absR *locus were able to produce Act normally when grown on MYM TE (data not shown), indicating that none were individually responsible for the reduction in Act production observed in the mutant strain.

### Other secondary metabolic gene clusters and processes are affected in the *relA *mutant strain

Bentley *et al. *[23] identified 21 genes or gene clusters in the genome of *S. coelicolor *predicted to specify for secondary metabolites. Analysis of the list of genes that were found to be significantly differently expressed between M570 (*relA*- ppGpp-) and M600 (*relA+ *ppGpp+) to identify pathways represented in the data indicated that, in addition to *act*, *red *and *whiE *mentioned above, clusters for coelichelin (*SCO0489*-*99*) the hopanoids (*SCO6759*-*71*), eicosapentaenoic acid (*SCO0124*-*29*), CDA (*SCO3210*-*49*), an unknown deoxysugar/glycosyltransferase product (*SCO0381*-*0401*), and an unknown type I polyketide synthase product (*SCO6273*-*88*) were affected, as illustrated in Figure S5 in Additional data file 7 (see also Additional data file 5 and Table S5 in Additional data file 3). Transcription of the *cda *antibiotic cluster had already been shown to be positively activated by ppGpp (see above), and it was perhaps surprising to find that only four *cda *cluster genes were apparently significantly affected in the ppGpp- mutant strain M570. However, inspection of the data for all 12 time points initially gathered suggested that the entire cluster was in fact influenced, with transcription being delayed by 6 h and significantly reduced in strain M570. This was confirmed by qRT-PCR analysis of representative genes from the cluster, which showed that in the 18 h samples, transcription of the regulator *cdaR *was two to three-fold higher in strain M600 in each biological replicate, and the gene encoding CDA peptide synthase I (*SCO3230*) was five- to eight-fold higher (data not shown). Transcription of the eicosapentaenoic acid (EPA) cluster appears to be temporally associated with sporulation, peaking at 60 h in the parent strain but not in the mutant. Nishida *et al*. [54] have shown that EPA can directly protect *E. coli *cells against oxidative damage by shielding the entry of reactive oxygen species, and it is possible that it provides similar protection for the spores of *S. coelicolor*.

Other functionally related sets of genes associated with secondary cellular processes that were significantly altered in expression in the mutant strain include one of the gene clusters annotated as being responsible for gas vesicle synthesis (*SCO0649*-*58 *[55]), and also the two sets of genes involved in carbon storage via production of glycogen/trehalose [56,57] (Figure S6 in Additional data file 7). Yeo and Chater [57] found that the *glgBI *genes (*SCO5440*-*44*) are responsible for glycogen deposition in vegetatively growing cells, whereas carbon storage in aerial and sporulating cells was performed by the *glgBII *cluster (*SCO7732*-*38*). In agreement with this, the profiles in Figure S6 (Additional data file 7) indicate that the *glgBI *genes are transcribed transiently during early growth of the parent strain M600, with expression of the *glgBII *cluster coinciding with morphological differentiation. In contrast, the mutant strain exhibits transcription of only the *glgBI *genes throughout its growth curve, consistent with the idea that it is unable to switch from vegetative to aerial growth on this media.

### Co-expressed sets of genes induced only in the *relA *mutant strain provide evidence that it is suffering from prolonged stress

**Figure 8:**
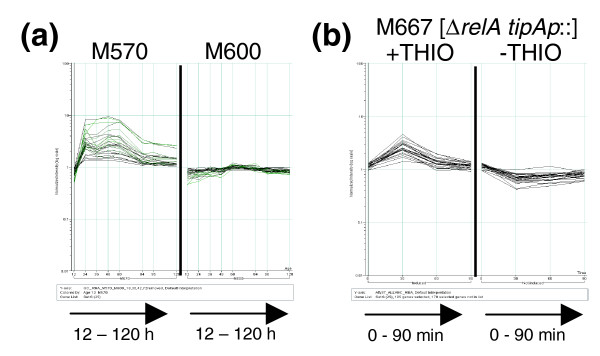
Genes in QT cluster 6 that were identified as being significantly over-expressed in **(a) **M570 (*relA*- ppGpp-) relative to M600 (*relA*+ ppGpp+) during growth on agar plates, are also induced **(b) **following addition of thiostrepton (THIO) to strain M667 [Δ*relA tipAp*::]. In each panel, the x-axis represents time (culture age in (a); time after induction in (b)), and the y-axis is normalized transcript abundance in log_10 _scale.

QT cluster analysis highlighted many sets of putatively co-expressed genes that were over-expressed at around 24 h in strain M570 (*relA*- ppGpp-) compared to the *relA*+ strain (Additional data file 6). Strikingly, the vast majority of the genes (55/59) from the four most populous clusters (QT6, 14, 27 and 42) were also found to be swiftly up-regulated following addition of thiostrepton to liquid grown *S. coelicolor *strain M667 [Δ*relA tipAp::*]. The data for the genes present in QT cluster 6 are presented in Figure 8 as an example. This suggests that after 24 h growth, the M570 culture begins to suffer a stress similar to that experienced by cells following addition of the antibiotic thiostrepton. Approximately 50% (26/55) of the affected genes are of completely unknown function, but 12 are annotated as GNAT-family acetyltransferases, and 6 are from ABC transport systems. Whether these are involved in alleviating stress by acetylating proteins or toxic metabolites, and exporting endogenously produced toxins from the cells remains to be determined.

The induction of ppGpp synthesis in liquid cultures of *S. coelicolor *was shown to influence ribosome biogenesis, certain transport systems, and a number of genes from major carbon metabolic pathways, amino acid metabolism and purine/pyrimidine biosynthesis (see above). A consideration of the pathways and functions represented by the genes found to be differentially expressed between the ppGpp- mutant M570 and the parental strain M600 indicates considerable differences in processes central to metabolism and transport (Table S5 in Additional data file 3, and also Additional data file 5). Significant alterations in the expression of genes involved in oxidative phosphorylation, amino acid biosynthesis and metabolism, the urea cycle, aminoacyl-tRNA synthesis, ubiquinone biosynthesis, and ribosome production were all observed, despite the fact that the mutant strain grew as rapidly as the parent during the early vegetative growth phase. The suggestion that strain M570 (*relA*- ppGpp-) was inducing some kind of stress response from 24 h onwards during growth is perhaps related to the disturbances in central metabolism, and may also be the root cause of the mutant strain's failure to proceed correctly through its developmental program under these growth conditions.

Strikingly, a total of 97 ABC transport system genes were significantly altered in expression in the mutant strain, plus 9 associated with protein export. The latter include six genes from the general Sec protein export system, which was found to be repressed by induction of ppGpp synthesis (see above), and one, *tatC*, from the TAT system responsible for exporting folded proteins [58]. The differentially expressed ABC transport systems included those predicted to be involved in translocation of oligopeptides, siderophores, amino acids, sugars and the RamAB transporter for export of the surfactant peptide SapB. Three genes encoding a putative sugar permease (*SCO3482-84*) are up-regulated in M600 from 60 h onwards, but not in the *relA *mutant. These are adjacent to *dagA *on the chromosome, encoding the secreted agarase enzyme [59], and may be involved in uptake of the products of agar degradation. They are clustered in their expression pattern with nine other genes from the same chromosomal locus, and an inverted repeat motif was identified upstream of four of these genes, suggesting a potential site for co-regulation (QT17 in Additional data file 6). Although M570 produces agarase normally on minimal media lacking sugars, *dagA *was not apparently expressed in the mutant strain on the MYMTE agar used for growth in this experiment, and agarase activity could not be detected (data not shown). Perturbations in transport systems required for correct nutrition could be envisaged to contribute to metabolic stress.

### Evidence for gross alterations in the regulation of the global transcriptional machinery associated with mutation of *relA*

*S. coelicolor *possesses 65 sigma factors capable of directing gene transcription via their ability to recruit RNA polymerase to specific sets of promoters. The mRNA abundance of 23 of these was significantly altered in the *relA *mutant strain M570, which, if translated into alterations in sigma factor protein abundance, could mediate wide-ranging effects on gene transcription. Of the four principal sigma factors σ-HrdA, B, C and D [30,60], transcription of *hrdD *was up-regulated in the mutant strain from 36 h to 60 h, while *hrdC *was higher in the parent in all but the earliest time point (Figure S7 in Additional data file 7). Transcription of *hrdA *was similar between the two strains (the gene for the major vegetative sigma factor *hrdB *is not represented on the GeneChip used). Interestingly, expression of the ECF-family sigma factor SigU (SCO2954) identified by Gehring *et al*. [61] as playing a role in morphological differentiation in *S. coelicolor *was massively up-regulated in the parent strain from 24 h onwards, but not at all in the mutant strain, which fails to differentiate. A group of 18 genes was identified by QT clustering that shared a similar expression profile (QT cluster 11 in Additional data file 6), and a common ECF sigma-like promoter sequence was found upstream of 8 of these genes, suggesting a regulon for SigU that includes transcription of its own promoter. Eleven genes reported to form part of the SigR regulon [62] were significantly differently expressed between the parent and mutant strain, with the abundance of mRNA from *sigR *itself being higher in the mutant strain between 24 and 60 h (Figure S7 in Additional data file 7). Transcription of one of the two *relA *promoters is at least partially dependent on SigR, suggesting a link with the ppGpp signaling mechanism. SigR transcribes the RNA polymerase binding protein RbpA, which, in contrast to the activity of ppGpp, exerts positive control over transcription from a rRNA promoter [63]. *rbpA *was significantly over-expressed in the ppGpp- mutant strain M570 from 24 h to 60 h (Figure S7 in Additional data file 7), perhaps suggesting that the mutant not only lacks the ability to stringently shut down rRNA synthesis, but is employing RbpA to actively stimulate rRNA production (and perhaps transcription of other genes) following the initial period of exponential growth.

### Genes involved in nitrogen metabolism are significantly over-expressed in strain M667 [Δ*relA tipAp::*] compared to M653 [Δ*relA tipAp::relA*(1.46 kb)]

A total of 428 genes were significantly differently expressed between non-induced cultures of strain M653 [Δ*relA tipAp::relA*(1.46 kb)], which exhibits a constitutively low level of ppGpp synthesis (about 6 pmol mg^-1 ^dry weight), and the control strain M667 [Δ*relA tipAp::*], which is totally defective in the ability to produce ppGpp (Additional data file 4). Interestingly, of the 352 genes that were more highly expressed in strain M667 (ppGpp = 0), those with functions related to nitrogen metabolism were significantly represented, and, in particular, transcription of glutamine synthase II (*glnII*) and the *amtB*-*glnK*-*glnD *operon was massively up-regulated (Figure S8 in Additional data file 7). This may reflect repression of these genes by the low levels of ppGpp present in strain M653 [Δ*relA tipAp::relA*(1.46 kb)], although increasing ppGpp levels from 6 to 20 pmol mg^-1 ^dry cell weight in the induction experiments had no significant affect on their expression. Another, perhaps more likely, possibility is that it is associated with the differing intracellular ATP concentrations between the two strains, where strain M667 [Δ*relA tipAp::*] has approximately 30% of the levels observed in M653 [Δ*relA tipAp::relA*(1.46 kb)] (Figure 1).

Genes from the antibiotic biosynthesis clusters Red, Act and CDA were also more highly expressed in strain M667 [Δ*relA tipAp::*] (Table S3 in Additional data file 3). The Act and CDA clusters were found to be activated following induction of ppGpp-synthesis in strain M653 [Δ*relA tipAp::relA*(1.46 kb)] (see above) and it seems paradoxical that a comparison of samples derived from cells with intracellular ppGpp concentrations measured at 0 or 6 pmol mg^-1 ^dry cell weight would show an over-expression of these clusters in the strain lacking ppGpp. However, the CDA cluster, although ultimately induced, appears to be repressed in the first 30 minutes after induction of ppGpp synthesis in M653, suggesting that it responds differently to subtly different concentrations of ppGpp, and this may also be the case for the other clusters. Indeed, different levels of ppGpp within the cells may create specific states of 'regulatory poise' that are reflected in different global patterns of gene transcription. The observation that 11 genes from two-component regulatory systems are significantly up-regulated in strain M667 (ppGpp = 0 pmol mg^-1^) compared to M653 (ppGpp = 6 pmol mg^-1^) supports this idea.

## Conclusion

If transcription of the *S. coelicolor *genome approximates to the situation described for *E. coli *by Bremer and Davies [64], then stable RNA synthesis probably constitutes about 80% of cellular transcription under optimal growth conditions. Ribosomal RNA synthesis, resulting from transcription of just six operons and directed by RNA polymerase containing the major vegetative sigma factor σ-HrdB, would account for 85% of this figure. Thus, the vast majority of cellular RNA polymerase during active growth would be concentrated into transcription foci centered on the rRNA operons in the nucleoid, analogous to observations made in *E. coli *[65,66]. This dominance of stable RNA synthesis is proposed to sequester most of the RNA polymerase during active growth, reducing its availability for transcription of other genes in the genome with functions non-essential for growth (reviewed in [67]). Induction of ppGpp synthesis in *E. coli *not only shuts down rRNA transcription, but also causes the transcription foci to rapidly disperse [65], thereby presumably freeing up RNA polymerase and facilitating the subsequent redirection of transcription, where genes important for starvation survival and virulence are favored over those required for growth and proliferation. The results of the present study portray the occurrence of an analogous process in *S. coelicolor*, where ppGpp synthesis causes a dramatic switch in cellular physiology, with transcription of genes more usually associated with stationary phase processes, including secondary metabolism and alternative ribosomal protein synthesis, being activated at the expense of those with functions important for active growth. This is presumably also additionally influenced by the observed significant decrease in transcription of the major vegetative sigma factor σ-*hrdB*, and possibly also by the corresponding induction of the alternative ECF sigma factor *SCO4005*.

*S. coelicolor *is a model organism for the streptomycetes, industrially important producers of bioactive secondary metabolites, and the observed ppGpp-mediated redirection of gene transcription involves changes in operons and gene clusters that are particularly characteristic of the genus: the repression of conservons, the induction of antibiotic gene clusters, and the expression of the morphogenetic *sapB*, chaplin and rodlin genes. While the exact regulatory route by which this is achieved remains to be determined (it may be a direct effect of ppGpp on the selection of the promoters of these genes by RNA polymerase), this study has revealed a number of genes with regulatory functions whose transcription is significantly altered following ppGpp synthesis, and has provided new insights and greatly advanced our understanding of the global regulatory influence of ppGpp in *S. coelicolor*.

## Materials and methods

### Bacterial strains

*S. coelicolor *M600 is a prototrophic plasmid-free derivative of *S. coelicolor *A3(2) [68]. M570 (Δ*relA*) is a mutant of M600 in which *relA *has been replaced by a hygromycin resistance cassette [7]. M653 [Δ*relA tipAp::relA*(1.46 kb)] and M667 [Δ*relA tipAp::*] are derivatives of M570 carrying the integrated plasmids pIJ6083 and pIJ8600, respectively [8]. M653 can be induced to produce ppGpp by treatment with thiostrepton, while M667 is a control strain that does not synthesise ppGpp following addition of thiostrepton.

### Culture conditions

For comparison of M600 and M570 (Δ*relA*) during surface growth on agar plates, NUNC bioassay dishes (245 × 245 × 25 mm) containing MYMTE agar (maltose 4 g l^-1^, yeast extract 4 g l^-1^, malt extract 10 g l^-1^, Difco Bacto agar 20 g l^-1^, R2YE trace element solution [68] 2 ml l^-1^) overlain with sterile cellophane were inoculated by evenly spreading 6 × 10^8 ^spores per plate, and incubated at 30°C. Samples of mycelia were harvested at 12, 18, 24, 30, 36, 42, 48, 60, 72, 84, 96 and 120 h by scraping off with a sterile spatula and immediately flash-frozen in liquid nitrogen. Cultures were visually assessed for morphological differentiation and production of pigmented antibiotics. Growth curves were performed in triplicate using spore preparations derived from independent single colonies (that is, biological triplicates), and samples were stored frozen at -80°C prior to RNA extraction.

The ppGpp induction experiments were performed using M653 and M667 essentially as described in Hesketh *et al. *[9]. Briefly, spores (about 10^10 ^cfu ml^-1^) were germinated in 2 xYT medium [68] for 7 h at 30°C. Germlings were harvested by centrifugation (5 minutes at 4000 g), resuspended in minimal medium supplemented with 0.2% Casamino acids (SMM) [68], and briefly sonicated to disperse any aggregates before inoculation into 50 ml SMM in 250 ml siliconized flasks containing coiled stainless steel springs. Each flask received the equivalent of 5 × 10^7 ^cfu, and flasks were incubated with vigorous agitation at 30°C. For each strain, when the OD450 nm reached 0.5-0.6, cultures were pooled to give 400 ml and the 0 minute sampling performed. The pooled culture was re-divided into two equal aliquots, and one half treated with 25 μg ml^-1 ^thiostrepton in DMSO to induce transcription from *tipAp *while the other half acted as the negative control and received only the equivalent volume of DMSO. Incubation was continued (approximately 180 ml volumes in 1 l flasks) and further samples were taken from both induced and control cultures after 30, 60 and 90 minutes. On sampling, 20 ml aliquots were taken for ppGpp assays, 10 ml for dry cell weight determination, and 10 ml for RNA extraction. The culture aliquot for producing the RNA sample was immediately treated with twice the volume of RNA protect bacteria solution (Qiagen, Germantown, MD, USA) according to the manufacturer's instructions to stabilize the RNA content of the cells, and the resultant cell pellet stored at -80°C prior to processing for RNA extraction. The experiments were performed in triplicate for each strain, using spore preparations derived from independent single colonies (that is, biological triplicates).

### RNA isolation and quality control

Total RNA was isolated from mycelia harvested and stored from liquid cultures using the RNeasy midi kit from Qiagen largely according to the manufacturer's instructions, but with several modifications. Cell pellets were resuspended in TE buffer (1 ml) containing 15 mg ml^-1 ^lysozyme and incubated for 60 minutes at room temperature. After addition of RNeasy RLT buffer (4 ml) samples were sonicated for 3 cycles of 20 s (Sanyo Soniprep 150, amplitude 18 microns), resting on ice for 1 minute between bursts, then extracted twice with phenol-chloroform (4 ml) and once with chloroform (4 ml). Extracts were then treated with ethanol (2.8 ml) and applied to the RNeasy midi columns for purification according to the supplied protocol, including an on-column DNaseI digestion step for 60 minutes at room temperature. Purified RNA was finally eluted in 300 μl RNase-free water. For purification of RNA from mycelia harvested from agar plates using RNeasy columns, cells were first disrupted by freeze-grinding under liquid nitrogen. Aliquots (0.1-0.25 g) of the frozen powder were transferred to a 2 ml Eppendorf tube containing RLT buffer (0.7 ml) and sterile sand (0.25 g: Sigma -50+70 mesh; Sigma, Gillingham, Dorset, UK), and vortexed vigorously for 90 s to shear genomic DNA and homogenize the sample. Cell debris and sand were removed by centrifugation (13,000 rpm, 4 minutes) and the sample mixed with ethanol (0.5 ml) then applied to an RNeasy mini column and processed according to the protocols supplied, including an on-column DNaseI digestion step for 60 minutes at room temperature. Purified RNA was finally eluted in 100 μl RNase-free water.

RNA concentration was determined by absorbance at 260 nm using a Nanodrop spectrophotometer, and absorbance ratios at 260/280 nm and 260/230 nm used to assess quality. All samples were also subjected to a final quality check by separating 1 μl of each using Agilent RNA6000 nano LabChips^® ^(Agilent Technology 2100 Bioanalyzer Version A.01.20 SI211; Agilent, Santa Clara, CA, USA).

### Affymetrix GeneChip hybridization and data collection

Purified total RNA (10 μg) was processed into labeled and fragmented cDNA for hybridization to *Streptomyces *diS_div712a GeneChip arrays according to the manufacturer's published protocol for *Pseudomonas aeruginosa *[69]. Briefly, cDNA synthesis was performed using 72% GC-content random primers and Superscript III reverse transcriptase (Invitrogen). The resulting cDNA was fragmented to approximately a 50-200 base-pair size range by partial digestion with DNaseI, and then terminally labeled with biotin using terminal transferase in the presence of biotinylated ddUTP. The labeled fragmented cDNA was hybridized to the GeneChips according to protocols provided by the manufacturer in a Hybridization Oven model 640 (Affymetrix, Santa Clara, CA, USA). The GeneChips were washed and stained with streptavidin-phycoerythrin using GeneChip fluidics workstation model 450, and then scanned with a Gene Array Scanner. Model 2500 was used for scanning the GeneChips from the M570 versus M600 comparison experiment, while all other GeneChips were scanned using model 3000 7G.

### General methods for Affymetrix GeneChip data analysis

The gene expression data were preprocessed using the Robust Multichip Average (RMA) algorithm of Irizarry *et al. *[70], as implemented in RMAExpress version 0.2. This performs steps for background adjustment, quantile normalization and summarization of the probe-level data to produce a single normalized value for expression of each gene on the chip. The data were then imported into GeneSpring 7.0 (Agilent Technologies, Santa Clara, California, USA), converting to log_2 _values and normalizing per gene to the median. Error models based on replicate values were implemented.

The quality of the array data was checked using a variety of tools, including the 'affyPLM', 'affy' and 'simpleaffy' packages for the statistical computing environment R [71], quality control methods available within GeneSpring 7, and data from report files generated in the Affymetrix GeneChip operating software following scanning of the arrays. Three arrays for the following samples failed quality control and were omitted from the analysis: non-induced sample at 60 minutes for replicate 1 of M667 [Δ*relA tipAp::*]; M600 growth curve 60 h sample replicate 2; M570 growth curve 60 h sample replicate 2.

Array data have been deposited at the MIAME-compliant ArrayExpress database under accession numbers E- MEXP-1098, E-MEXP-1119 and E-MEXP-1120 [72].

### Identification and analysis of significantly differentially expressed genes

Typically for each experiment detailed below, the quality controlled array data were filtered in GeneSpring to remove genes deemed to be expressed at a level below reliable detection by determining those with a raw signal value below a defined background cut-off value (usually 20 or 25) in all samples. The results were further filtered to remove genes not significantly changing under the conditions of the experiment by identifying those with normalized expression values between 0.8 and 1.2 (1.5-fold change limit) or 0.667 and 1.334 (2-fold change limit) in all conditions, as stated in the text. The filtered data were then subjected to two-way ANOVA to identify genes significantly altered under the experimental conditions. The two parameters tested were 'induction' and 'time' for the thiostrepton induction experiments, and 'strain' and 'time' for the growth curve comparison of strains M570 and M600. Two-way ANOVA is able to assess the individual effect on transcript abundance of each parameter in each sample, and also whether there is an interaction or additive effect between the parameters. Three probability values (P values) are generated: one for each parameter independently, and one measuring the interaction between the two parameters. Two-way ANOVA was performed in GeneSpring using the parametric test option with a false discovery rate of *P *< 0.01 or *P *< 0.05, and assuming variances to be equal. *P *values were corrected using the Benjamini and Hochberg false discovery rate multiple testing correction procedure. Details of the statistical calculations used in the software can be accessed through the manufacturer's manual.

Final lists of significantly differently expressed genes were analysed to identify over-represented (*P *< 0.05) pathways or functions using the 'biological pathway analysis' script in GeneSpring, cross-comparing the gene lists to the pathways listed in KEGG [73] and to in-house collated lists of secondary metabolism clusters, and functional groups of genes. To identify groups of genes exhibiting similar expression profiles, QT clustering was performed in GeneSpring with appropriate user-defined correlation coefficient cut-off values. Where mentioned, the upstream regions of co-expressed genes were analysed for common promoter elements using the MEME DNA motif search tool [74].

#### Upon induction of ppGpp synthesis in M653 [Δ*relA tipAp::relA*(1.46 kb)]

Quality controlled data for genes were filtered to remove those with raw signal values below 20 in all samples (992 genes removed), and with normalized expression values between 0.8 and 1.2 in all 8 conditions (3,962 more genes removed). The remaining list of 2,703 genes was subjected to two-way ANOVA (*P *< 0.05) to identify those genes significantly differentially expressed between the induced and non-induced cultures.

A final list of ppGpp-affected genes was produced by removing those determined from the control experiment using strain M667 [Δ*relA tipAp::*] as being thiostrepton-affected (see below). This was divided into clearly ppGpp-repressed and ppGpp-induced genes as follows. For each of the 30, 60 or 90 minute induced samples, those genes with normalized expression values >1.5-fold lower or higher than both the 0 minute sample and the non-induced sample taken at the corresponding time were identified, and the data for each time point combined.

#### Changes in gene expression in M667 [Δ*relA tipAp::*] upon treatment with thiostrepton

Quality controlled data for genes (omitting the 60 minute sample for which a full set of data was not available) were filtered to remove those with raw signal values below 20 in all samples (448 genes removed), and with normalized values between 0.8 and 1.2 in all 8 conditions (3,685 more genes removed). The remaining list of 3,524 genes was subjected to two-way ANOVA (*P *< 0.05) to identify those genes significantly affected by thiostrepton addition. Only four genes were obtained using this approach (Additional data file 8), which is clearly an underestimate, and likely to reflect the fact that the analyses were performed only in duplicate for this study, rather than triplicate as above, thus reducing the penetration of the statistical analysis. To identify additional genes induced or repressed by thiostrepton addition, the 30 and 90 minute sample data were used to determine those genes that are 1.5-fold or higher up- or down-regulated in expression over both the 0 minute sample and the non-induced sample taken at the corresponding time. This yielded 172 and 258 genes for the 30 and 90 minute datasets, respectively, and a combined list of 416 thiostrepton-affected genes that includes the four genes previously identified as being significantly differentially expressed (Additional data file 8). Comparison with the 752 genes identified as being significantly affected by thiostrepton-induction of ppGpp synthesis in strain M653 [Δ*relA tipAp::relA*(1.46 kb)] revealed a total of 163 genes that appear in both lists and whose expression can therefore be considered as being reproducibly altered by addition of thiostrepton.

#### Changes in gene expression between non-induced samples of strain M653 and M667

The microarray data for the 0, 30 and 90 minute non-induced samples of strains M653 and M667 were filtered to remove those with raw signal values below 25 in all samples (956 genes removed), and with normalized values between 0.667 and 1.334 in all conditions (4,401 more genes removed). The remaining list of 2,298 genes was subjected to two-way ANOVA (*P *< 0.01) to identify those genes significantly different between the two strains.

#### Changes in gene expression between M600 (*relA*+) and M570 (*relA*-) during growth on MYMTE

The microarray data for time points 12, 24, 36, 48, 60, 84, 96, and 120 h were filtered to remove those with raw signal values below 25 in all samples (2,882 genes removed), and with normalized values between 0.667 and 1.334 in all conditions (1,558 more genes removed). The remaining list of 3,217 genes was subjected to two-way ANOVA (*P *< 0.01) to identify those genes significantly differently expressed between the two strains.

### Quantification of intracellular nucleotides

Extraction and HPLC analysis of ppGpp, ATP and GTP was carried out as described by Strauch *et al. *[16].

### qRT-PCR analysis of selected differentially expressed genes

Specific primers for selected genes of interest were designed using the web-based tool Primer3 [75]. Each RNA sample (5 μg) was subjected to RNase-free DNaseI treatment (Invitrogen (Carlsbad, CA, USA), amplification grade) in a 50 μl reaction volume according to the manufacturer's instructions. For cDNA synthesis, 8 μl of the resulting DNaseI-treated RNA was used as template in a 20 μl reaction volume employing Superscript III First Strand Synthesis Supermix (Invitrogen) according to the manufacturer's instructions. PCR cycling was performed at 25°C for 10 minutes, 42°C for 120 minutes, 50°C for 30 minutes, 55°C for 30 minutes and then 5 minutes at 85°C. To control for DNA contamination inthe qRT-PCR, a duplicate set of cDNA synthesis reactions were performed but with the reverse transcriptase enzyme omitted. Following RNaseH treatment, the samples were diluted 1:100 with Tris-EDTA (10 mM to 1 mM, pH 8.0), and 2.5 μl were used in the quantitative PCR reaction with SYBR Greener qPCR supermix (Invitrogen) according to the manufacturer's instructions. Each 25 μl reaction contained 200 nM of forward and reverse primers, and 3 μl of 40% DMSO. PCR cycling was performed in a Chromo4 machine (BioRad, CA, USA), typically at 50°C for 2 minutes, 95°C for 10 minutes, followed by 40 cycles of 95°C for 15 s and 58°C for 60 s. Parallel reactions were performed in the same 96-well plate using different dilutions of genomic DNA to generate a standard curve for each selected gene. All determinations were performed in triplicate, and the results were analysed using Opticon 2 Monitor software (MJ Research, Waltham, MA, USA). All values were ultimately normalized to an endogenous control gene, *SCO4742*. This gene was selected from the microarray data following a search for genes whose expression was unaffected by induction of ppGpp synthesis, by mutation in *relA*, and by sampling time. The control samples from cDNA synthesis lacking reverse transcriptase gave values comparable to background in all cases, indicating that the RNA samples were not contaminated with genomic DNA.

### Detection of SapB

SapB extraction and detection were performed according to Willey *et al. *[76], using Western blots visualized with the Amersham ECL Western Blotting detection system (GE Healthcare, Uppsala, Sweden) according to the manufacturer's instructions. Biological duplicate cultures were extracted after 48 h growth on MYMTE, and both sets gave similar results.

## Additional data files

The following additional data are available with the online version of this paper. Additional data file 1 lists the changes in gene expression upon induction of ppGpp synthesis in M653 [Δ*relA tipAp::relA*(1.46 kb)]. Additional data file 2 lists genes whose expression is significantly affected by ppGpp. Additional data file 3 contains supplementary Tables S1-S5. Additional data file 4 lists changes in gene expression between non-induced samples of strain M653 [Δ*relA tipAp::relA*(1.46 kb)] and M667 [Δ*relA tipAp::*]. Additional data file 5 lists significantly differently expressed genes between strain M600 (*relA*+ ppGpp+) and M570 (*relA*- ppGpp-) during growth on MYM TE agar. Additional data file 6 summarises the results of QT clustering of 2,031 genes significantly differently expressed between M600 (*relA*+ ppGpp+) and M570 (*relA*- ppGpp-). Additional data file 7 contains supplementary Figures S1-S8. Additional data file 8 shows changes in gene expression in M667 [Δ*relA tipAp*::] upon treatment with thiostrepton.

## Supplementary Material

Additional data file 1 Lists the results of two-way ANOVA of the microarray data obtained following the induction of M653 [Δ*relA tipAp::relA*(1.46 kb)] cultures by treatment with thiostrepton. Click here for file

Additional data file 2 Genes whose expression is significantly affected by ppGpp Click here for file

Additional data file 3 The tables summarise the pathways and processes that are significantly represented by the 189 genes shown to be ppGpp-repressed (Table S1); the 98 genes shown to be ppGpp-induced (Table S2); the 352 genes more highly expressed in non-induced cultures of M667 compared to M653 (Table S3); the 76 genes reduced in expression in non-induced cultures of M667 compared to M653 (Table S4); and the 2031 genes significantly differently expressed in M570 compared to M600 (Table S5). Click here for file

Additional data file 4 Lists the results of two-way ANOVA of the microarray data obtained when comparing the non-induced cultures of strains M653 and M667 Click here for file

Additional data file 5 Lists the results of two-way ANOVA of the microarray data obtained when comparing strains M600 (*relA*+ ppGpp+) and M570 (*relA*- ppGpp-) during surface growth on solid media Click here for file

Additional data file 6 QT clustering of 2031 genes significantly differently expressed between M600 (*relA*+ ppGpp+) and M570 (*relA*- ppGpp-) Click here for file

Additional data file 7 Figures S1-S4 present qRT-PCR data quantifying expression of genes following induction of ppGpp synthesis: S1 shows cvn1, cvn10 and cvn13; S2 shows *actII-ORF4 *and *cdaR*; S3 shows SCO4198 and SCO4336; and S4 shows SCO6264. Figures S5-S7 display expression profiles for genes that are significantly differently expressed between M600 and M570: S5 shows secondary metabolite gene clusters; S6 shows glycogen biosynthesis clusters and the *gvp2 *cluster; and S7 shows *hrdC*, *hrdD*, *sigR *and *rbpA*. Figure S8 compares expression profiles of *glnII*, *amtB*, *glnK *and *glnD *in non-induced cultures of M667 and M653. Click here for file

Additional data file 8 Lists genes in *S. coelicolor *whose expression was identified as being affected by thiostrepton (as detailed in the Materials and methods) Click here for file
