# Supplementary material for: The global role of ppGpp synthesis in morphological differentiation and antibiotic production in Streptomyces coelicolor A3(2)
Source: Genome Biol. 2007 Aug 3;8(8):R161. doi: 10.1186/gb-2007-8-8-r161 (PMC2374992; doi:10.1186/gb-2007-8-8-r161)
Supplement: Additional data file 3 — The tables summarise the pathways and processes that are significantly represented by the 189 genes shown to be ppGpp-repressed (Table S1); the 98 genes shown to be ppGpp-induced (Table S2); the 352 genes more highly expressed in non-induced cultures of M667 compared to M653 (Table S3); the 76 genes reduced in expression in non-induced cultures of M667 compared to M653 (Table S4); and the 2031 genes significantly differently expressed in M570 compared to M600 (Table S5). [file gb-2007-8-8-r161-S3.doc]

Table S1. Pathways or processes significantly represented in the 189 genes shown to be ppGpp-repressed. Cross-comparisons with a) the KEGG Streptomyces coelicolor pathways database, b) secondary metabolite gene clusters, and c) in-house collated list of genes with commonality of function, are shown.

| Pathway/cluster/function | Number of common genes with each pathway | GeneList vs Pathway  random overlap p-value  (P<0.05 are shown) |
| --- | --- | --- |
| A) KEGG Ribosome | 14 | 1.99E-11 |
| Reductive carboxylate cycle (CO2 fixation) | 8 | 1.27E-07 |
| ATP synthesis | 5 | 4.58E-07 |
| Butanoate metabolism | 9 | 2.94E-06 |
| Citrate cycle (TCA cycle) | 7 | 6.29E-06 |
| Oxidative phosphorylation | 9 | 6.71E-06 |
| Porphyrin and chlorophyll metabolism | 6 | 7.57E-05 |
| C5-Branched dibasic acid metabolism | 3 | 0.000143 |
| Peptidoglycan biosynthesis | 4 | 0.000397 |
| Benzoate degradation via CoA ligation | 5 | 0.000538 |
| Protein export | 4 | 0.00155 |
| Propanoate metabolism | 5 | 0.00284 |
| Purine metabolism | 6 | 0.00598 |
| Sulfur metabolism | 2 | 0.0117 |
| Lysine degradation | 3 | 0.0205 |
| Valine, leucine and isoleucine degradation | 4 | 0.021 |
| Glutathione metabolism | 2 | 0.0239 |
| One carbon pool by folate | 2 | 0.0288 |
| Pentose phosphate pathway | 3 | 0.047 |
|  |  |  |
| B) Secondary metabolite clusters |  |  |
|  |  |  |
| CDA | 5 | 0.00575 |
|  |  |  |
| C) In-house list of genes with common function |  |  |
|  |  |  |
| 50S ribosomal proteins | 8 | 0.00000158 |
| 30S ribosomal proteins | 6 | 0.00000303 |
| Conservons | 11 | 0.00000349 |
| Cobolamin riboswitch | 6 | 0.000299 |
| Vitamin B12 | 3 | 0.00211 |
| Sec protein secretion system | 2 | 0.00852 |
| SigR-regulon | 3 | 0.0254 |
|  |  |  |

Table S2. Pathways or processes significantly represented in the 98 genes shown to be ppGpp-induced. Cross-comparisons with a) the KEGG Streptomyces coelicolor pathways database, b) secondary metabolite gene clusters, and c) in-house collated list of genes with commonality of function, are shown.

| Pathway/cluster/function | Number of common genes with each pathway | GeneList vs Pathway  random overlap p-value  (P<0.05 are shown) |
| --- | --- | --- |
| A) KEGG Biosynthesis of type II polyketide products | 3 | 0.000311 |
| Inositol phosphate metabolism | 2 | 0.00432 |
| Fatty acid biosynthesis | 2 | 0.0265 |
| Reductive carboxylate cycle (CO2 fixation) | 2 | 0.0432 |
|  |  |  |
| B) Secondary metabolite clusters |  |  |
|  |  |  |
| CDA | 23 | 2.34E-32 |
| Act | 5 | 0.00000869 |
| Hopanoids | 2 | 0.0115 |
|  |  |  |
| C) In-house list of genes with common function |  |  |
|  |  |  |
| SigR-regulon | 2 | 0.0432 |
|  |  |  |

Table S3. Pathways or processes significantly represented in the 352 genes significantly more highly expressed in non-induced cultures of strain M667 (ppGpp = 0 pmol mg^-1^) than M653 (ppGpp = 6 pmol mg^-1^). Cross-comparisons with a) the KEGG Streptomyces coelicolor pathways database, b) secondary metabolite gene clusters, and c) in-house collated list of genes with commonality of function, are shown.

| Pathway/cluster/function | Number of common genes with each pathway | GeneList vs Pathway  random overlap p-value  (P<0.05 are shown) |
| --- | --- | --- |
| A) KEGG Two-component regulatory systems | 11 | 0.00000808 |
| Nitrogen metabolism | 8 | 0.0000338 |
| Biosynthesis of type II polyketide products | 5 | 0.0000733 |
| ABC transporters - General | 34 | 0.000119 |
| Phenylalanine, tyrosine and tryptophan biosynthesis | 7 | 0.00107 |
| Fatty acid biosynthesis | 4 | 0.0118 |
|  |  |  |
| B) Secondary metabolite clusters |  |  |
|  |  |  |
| CDA | 38 | 1.94E-43 |
| Red | 13 | 0.00000000000248 |
| Coelichelin | 9 | 0.000000000162 |
| Act | 7 | 0.0000529 |
| Cesferrioxamines | 2 | 0.0119 |
|  |  |  |
| C) In-house list of genes with common function |  |  |
|  |  |  |
| Gas vesicle cluster 1 | 4 | 0.000265 |
| Gln genes | 4 | 0.000265 |
| Phosphate transport/regulation | 3 | 0.0121 |
| Conservons | 6 | 0.0285 |
| Cobalamin riboswitch | 5 | 0.0288 |
| GNAT-family acetlytransferases | 5 | 0.041 |
|  |  |  |

Table S4. Pathways or processes significantly represented in the 76 genes significantly more lowly expressed in non-induced cultures of strain M667 (ppGpp = 0 pmol mg^-1^) than M653 (ppGpp = 6 pmol mg^-1^). Cross-comparisons with a) the KEGG Streptomyces coelicolor pathways database, b) secondary metabolite gene clusters, and c) in-house collated list of genes with commonality of function, are shown.

| Pathway/cluster/function | Number of common genes with each pathway | GeneList vs Pathway  random overlap p-value  (P<0.05 are shown) |
| --- | --- | --- |
| A) KEGG Glutathione metabolism | 2 | 0.00416 |
| Arachidonic acid metabolism | 1 | 0.0391 |
|  |  |  |
| B) Secondary metabolite clusters |  |  |
|  |  |  |
| None |  |  |
|  |  |  |
| C) In-house list of genes with common function |  |  |
|  |  |  |
| GNAT-family acetyltransferases | 3 | 0.032 |
| TTA-containing genes | 4 | 0.0438 |
|  |  |  |

Table S5. Pathways or processes significantly represented in the 2031 genes shown to be significantly altered in expression between M600 and M570. Cross-comparisons with a) the KEGG Streptomyces coelicolor pathways database, b) secondary metabolite gene clusters, and c) in-house collated list of genes with commonality of function, are shown.

| Pathway/cluster/function | Number of common genes with each pathway | GeneList vs Pathway  random overlap p-value  (P<0.05 are shown) |
| --- | --- | --- |
| A) KEGG Biosynthesis of type II polyketide products | 10 | 0.0000142 |
| Urea cycle and metabolism of amino groups | 13 | 0.000158 |
| Biosynthesis of type II polyketide backbone | 6 | 0.000346 |
| Aminoacyl-tRNA biosynthesis | 13 | 0.00126 |
| Oxidative phosphorylation | 25 | 0.00186 |
| Ubiquinone biosynthesis | 14 | 0.00662 |
| Arginine and proline metabolism | 15 | 0.00734 |
| Valine, leucine and isoleucine biosynthesis | 13 | 0.0129 |
| C5-Branched dibasic acid metabolism | 4 | 0.0195 |
|  |  |  |
| B) Secondary metabolite clusters |  |  |
|  |  |  |
| Act | 22 | 0.00000000000331 |
| Coelichelin | 12 | 0.000000118 |
| Deoxysugar-glycosyltransferase | 17 | 0.000000289 |
| WhiE polyketide spore pigment | 8 | 0.0000243 |
| Red | 15 | 0.000111 |
|  |  |  |
| C) In-house list of genes with common function |  |  |
|  |  |  |
| Chaplins and rodlins | 9 | 0.0000489 |
| Gas vesicle cluster 2 | 9 | 0.0000489 |
| Sec protein secretion system | 6 | 0.000346 |
| Ram cluster | 5 | 0.00131 |
| Vitamin B12 biosynthesis | 8 | 0.0018 |
| Nar3 nitrate reductase | 4 | 0.00494 |
| tRNA synthetases | 14 | 0.00662 |
| Conservons | 21 | 0.0159 |
| Carbon storage glgBI cluster | 4 | 0.0195 |
| wbl genes | 6 | 0.0263 |
| Cobalamin riboswitch | 16 | 0.0266 |
| 30S ribosomal protein | 9 | 0.0281 |
