# Supplementary material for: The global role of ppGpp synthesis in morphological differentiation and antibiotic production in Streptomyces coelicolor A3(2)
Source: Genome Biol. 2007 Aug 3;8(8):R161. doi: 10.1186/gb-2007-8-8-r161 (PMC2374992; doi:10.1186/gb-2007-8-8-r161)
Supplement: Additional data file 7 — Figures S1-S4 present qRT-PCR data quantifying expression of genes following induction of ppGpp synthesis: S1 shows cvn1, cvn10 and cvn13; S2 shows actII-ORF4 and cdaR; S3 shows SCO4198 and SCO4336; and S4 shows SCO6264. Figures S5-S7 display expression profiles for genes that are significantly differently expressed between M600 and M570: S5 shows secondary metabolite gene clusters; S6 shows glycogen biosynthesis clusters and the gvp2 cluster; and S7 shows hrdC, hrdD, sigR and rbpA. Figure S8 compares expression profiles of glnII, amtB, glnK and glnD in non-induced cultures of M667 and M653. [file gb-2007-8-8-r161-S7.ppt]

## Slide 1
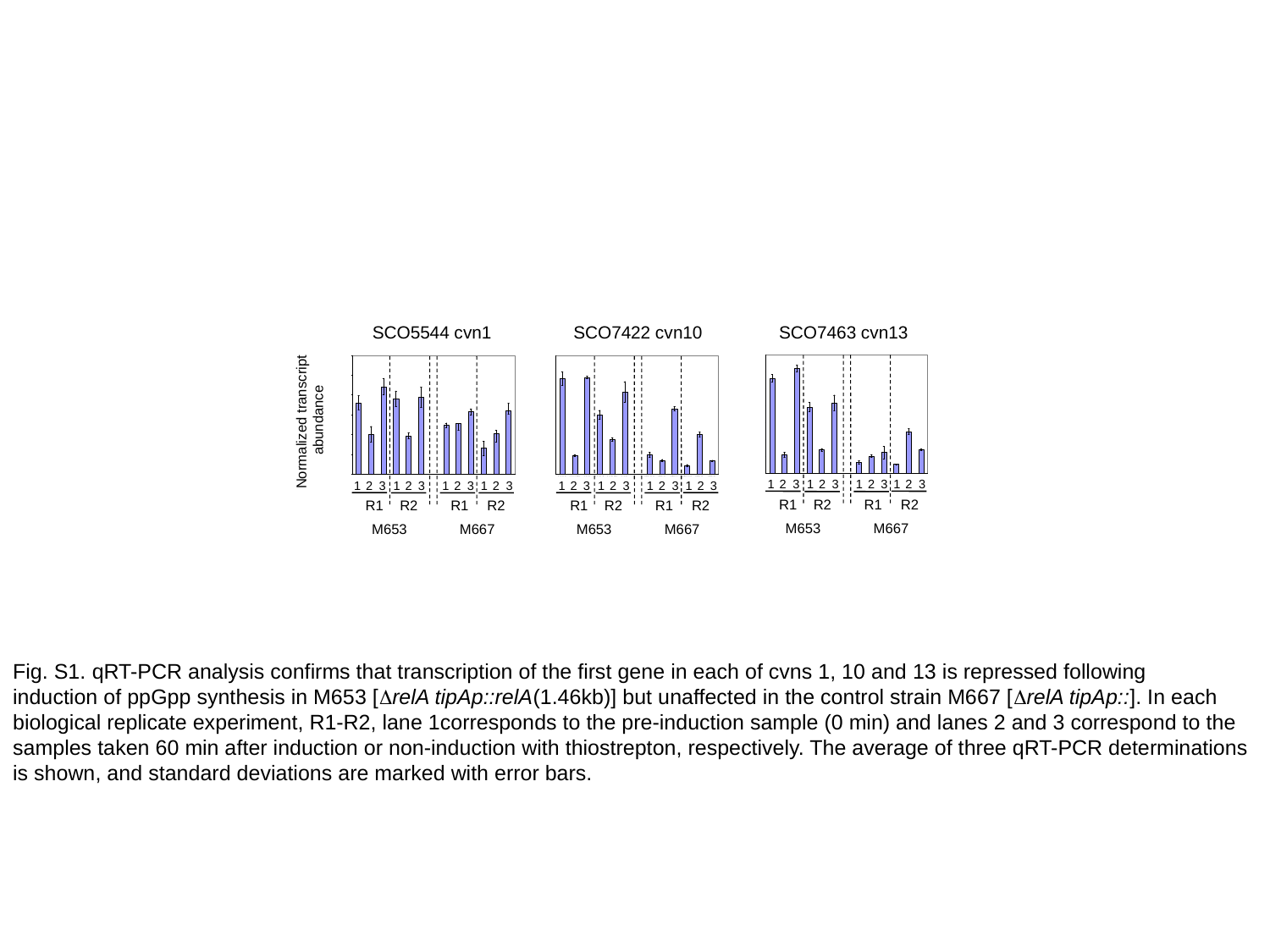

SCO5544 cvn1
SCO7422 cvn10
SCO7463 cvn13
Normalized transcript
abundance
1
 2
 3
1
 2
 3
1
 2
 3
1
 2
 3
1
 2
 3
1
 2
 3
1
 2
 3
1
 2
 3
1
 2
 3
1
 2
 3
1
 2
 3
1
 2
 3
R1
R2
R1
R2
R1
R2
R1
R2
R1
R2
R1
R2
M653
M667
M653
M667
M653
M667
Fig. S1. qRT-PCR analysis confirms that transcription of the first gene in each of cvns 1, 10 and 13 is repressed following
induction of ppGpp synthesis in M653 [ΔrelA tipAp::relA(1.46kb)] but unaffected in the control strain M667 [ΔrelA tipAp::]. In each
biological replicate experiment, R1-R2, lane 1corresponds to the pre-induction sample (0 min) and lanes 2 and 3 correspond to the
samples taken 60 min after induction or non-induction with thiostrepton, respectively. The average of three qRT-PCR determinations
is shown, and standard deviations are marked with error bars.

## Slide 2
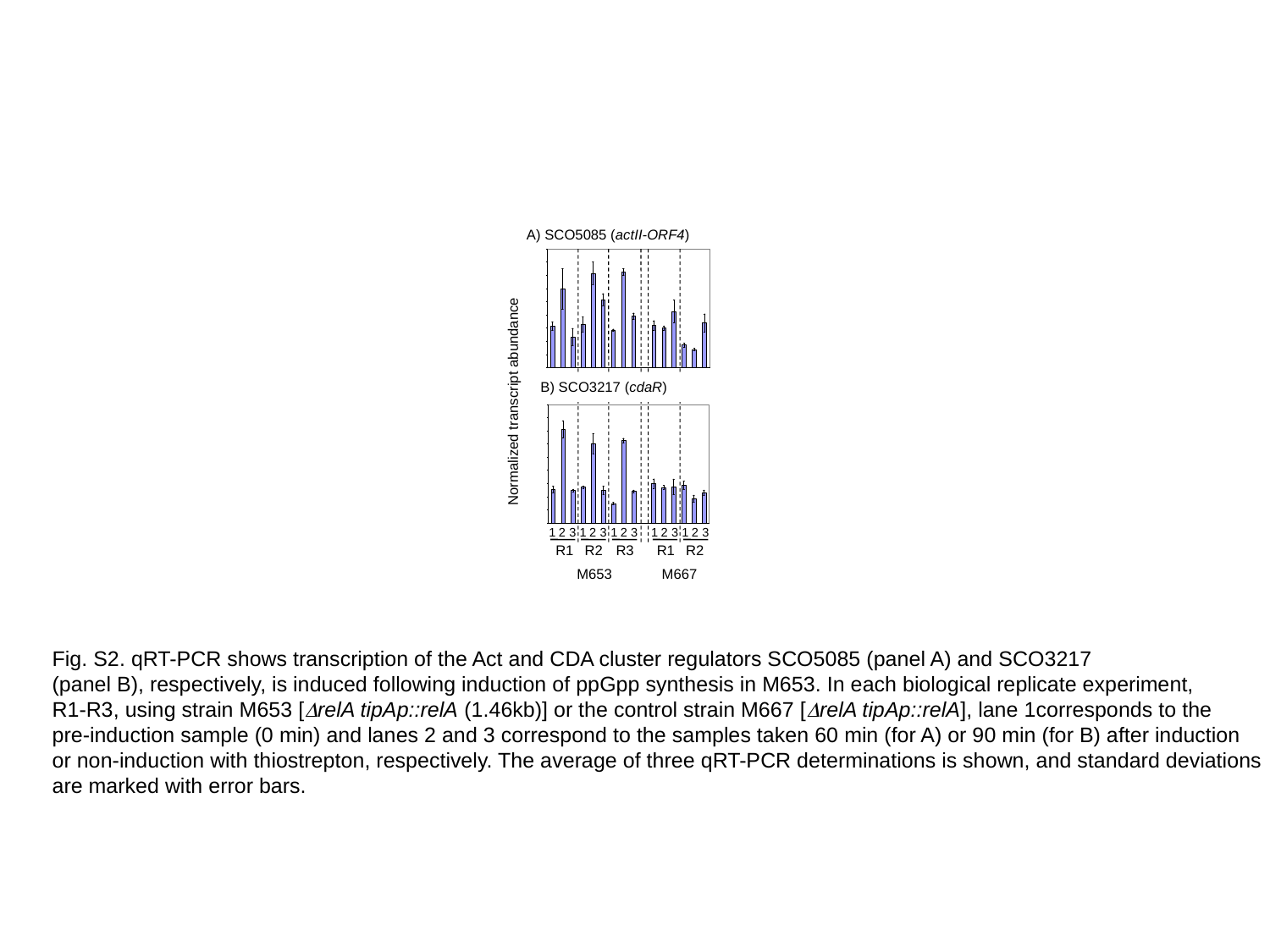

A) SCO5085 (actII-ORF4)
B) SCO3217 (cdaR)
Normalized transcript abundance
1
2
3
1
2
3
1
2
3
1
2
3
1
2
3
R1
R2
R3
R1
R2
M653
M667
Fig. S2. qRT-PCR shows transcription of the Act and CDA cluster regulators SCO5085 (panel A) and SCO3217
(panel B), respectively, is induced following induction of ppGpp synthesis in M653. In each biological replicate experiment,
R1-R3, using strain M653 [ΔrelA tipAp::relA (1.46kb)] or the control strain M667 [ΔrelA tipAp::relA], lane 1corresponds to the
pre-induction sample (0 min) and lanes 2 and 3 correspond to the samples taken 60 min (for A) or 90 min (for B) after induction
or non-induction with thiostrepton, respectively. The average of three qRT-PCR determinations is shown, and standard deviations
are marked with error bars.

## Slide 3
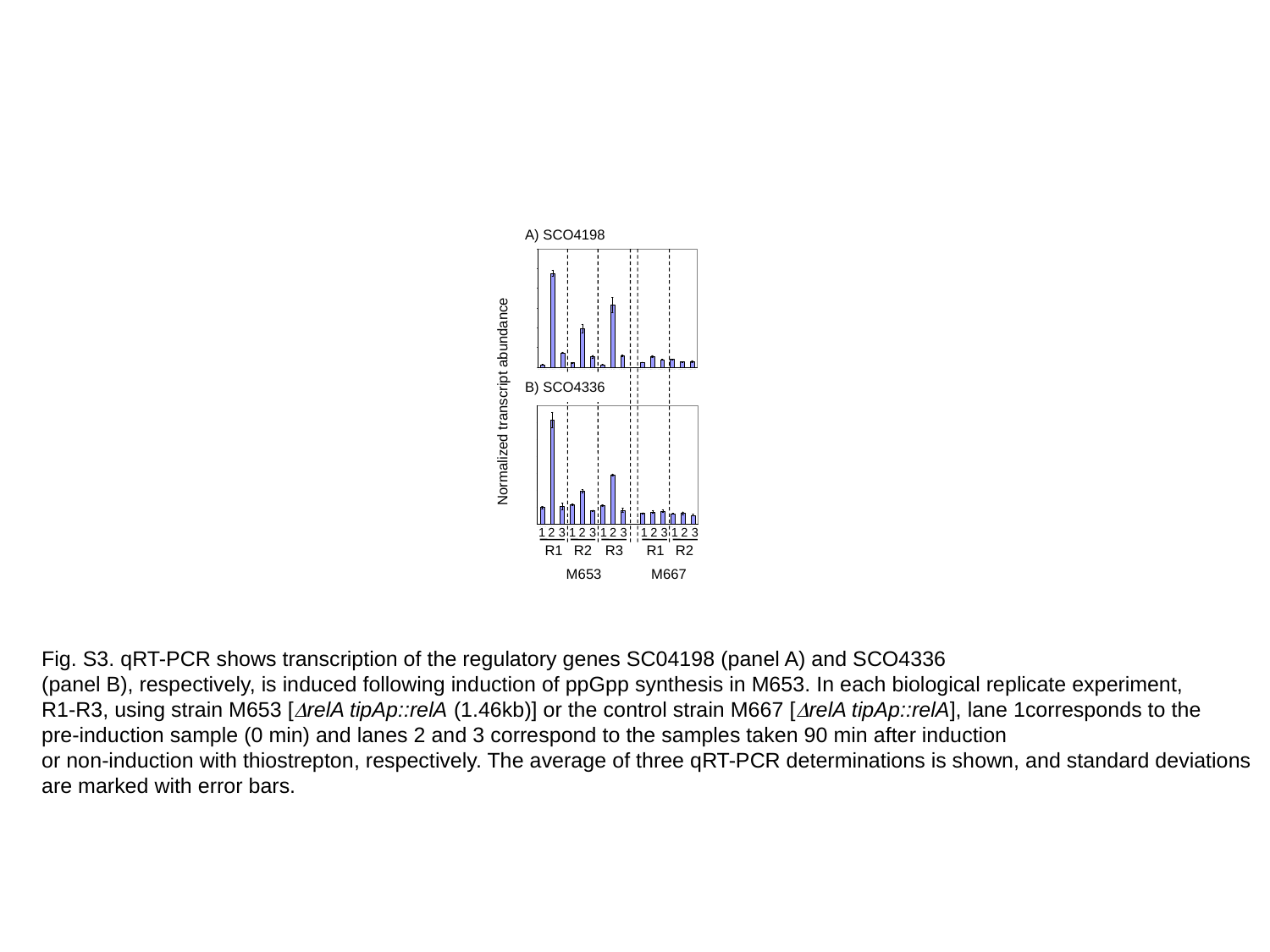

A) SCO4198
B) SCO4336
Normalized transcript abundance
1
2
3
1
2
3
1
2
3
1
2
3
1
2
3
R1
R2
R3
R1
R2
M653
M667
Fig. S3. qRT-PCR shows transcription of the regulatory genes SC04198 (panel A) and SCO4336
(panel B), respectively, is induced following induction of ppGpp synthesis in M653. In each biological replicate experiment,
R1-R3, using strain M653 [ΔrelA tipAp::relA (1.46kb)] or the control strain M667 [ΔrelA tipAp::relA], lane 1corresponds to the
pre-induction sample (0 min) and lanes 2 and 3 correspond to the samples taken 90 min after induction
or non-induction with thiostrepton, respectively. The average of three qRT-PCR determinations is shown, and standard deviations
are marked with error bars.

## Slide 4
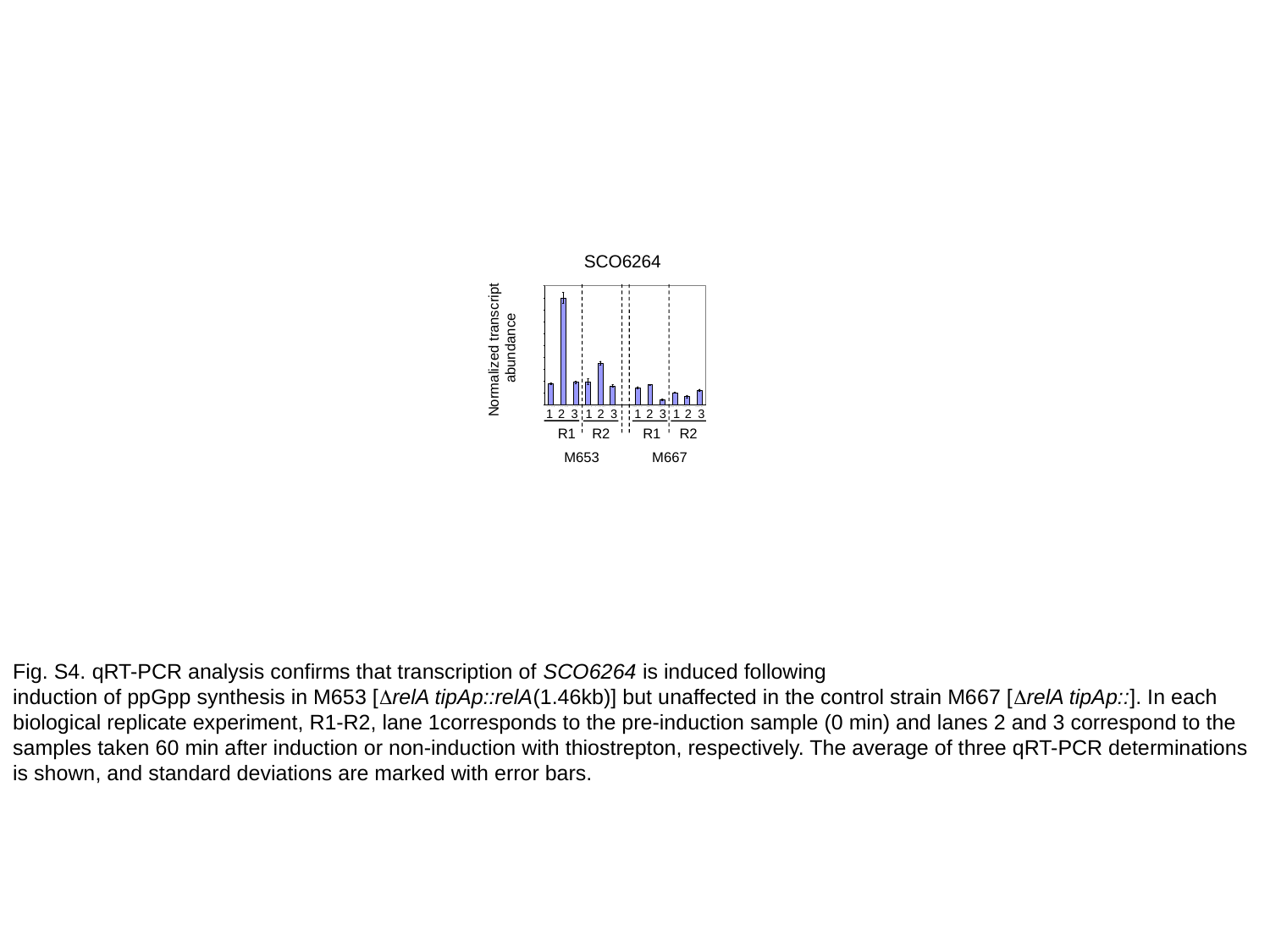

SCO6264
Normalized transcript
abundance
1
 2
 3
1
 2
 3
1
 2
 3
1
 2
 3
R1
R2
R1
R2
M653
M667
Fig. S4. qRT-PCR analysis confirms that transcription of SCO6264 is induced following
induction of ppGpp synthesis in M653 [ΔrelA tipAp::relA(1.46kb)] but unaffected in the control strain M667 [ΔrelA tipAp::]. In each
biological replicate experiment, R1-R2, lane 1corresponds to the pre-induction sample (0 min) and lanes 2 and 3 correspond to the
samples taken 60 min after induction or non-induction with thiostrepton, respectively. The average of three qRT-PCR determinations
is shown, and standard deviations are marked with error bars.

## Slide 5
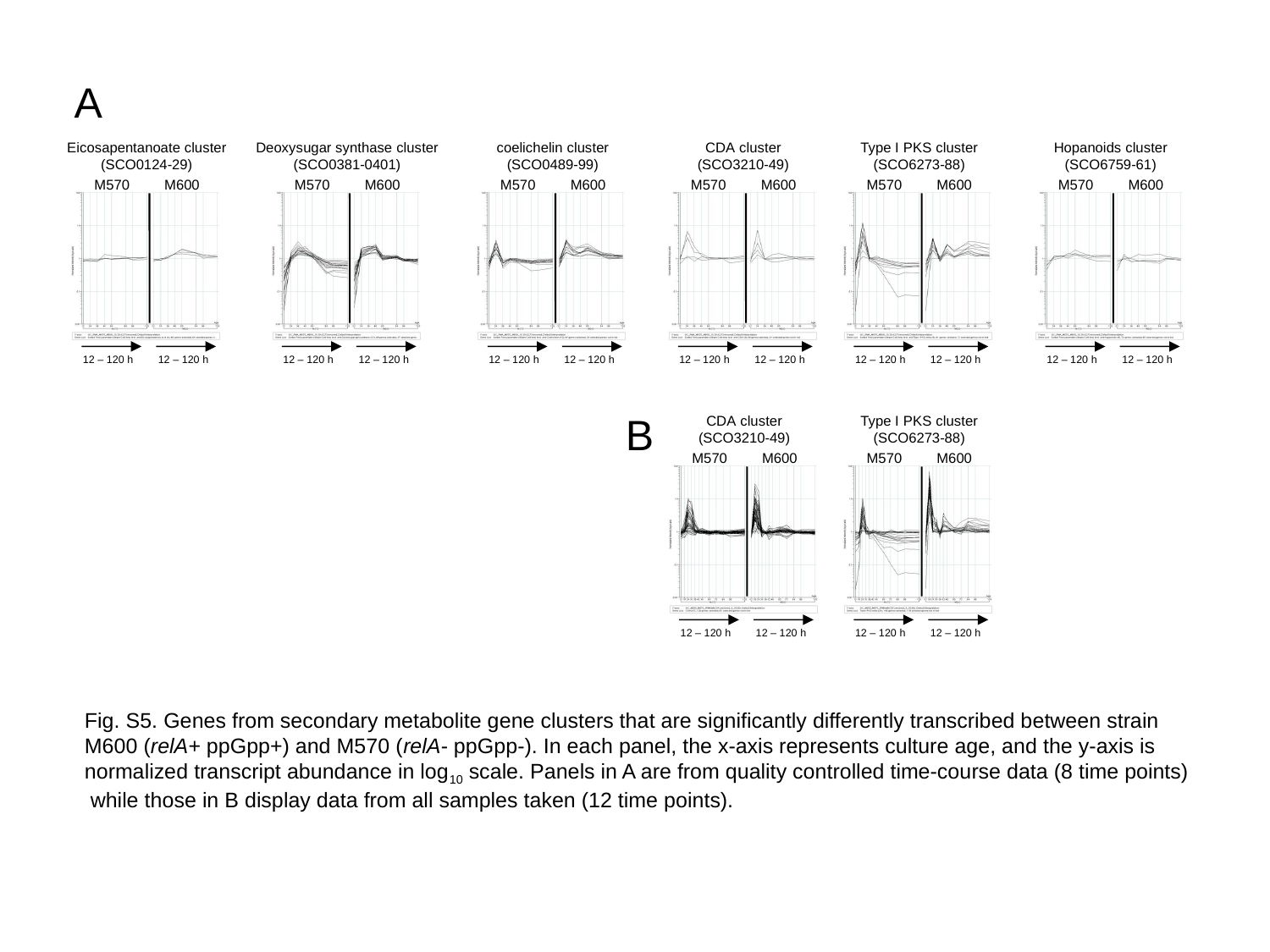

A
Eicosapentanoate cluster
(SCO0124-29)
M570
M600
12 – 120 h
12 – 120 h
Deoxysugar synthase cluster
(SCO0381-0401)
M570
M600
12 – 120 h
12 – 120 h
coelichelin cluster
(SCO0489-99)
M570
M600
12 – 120 h
12 – 120 h
CDA cluster
(SCO3210-49)
M570
M600
12 – 120 h
12 – 120 h
Type I PKS cluster
(SCO6273-88)
M570
M600
12 – 120 h
12 – 120 h
Hopanoids cluster
(SCO6759-61)
M570
M600
12 – 120 h
12 – 120 h
B
CDA cluster
(SCO3210-49)
M570
M600
12 – 120 h
12 – 120 h
Type I PKS cluster
(SCO6273-88)
M570
M600
12 – 120 h
12 – 120 h
Fig. S5. Genes from secondary metabolite gene clusters that are significantly differently transcribed between strain
M600 (relA+ ppGpp+) and M570 (relA- ppGpp-). In each panel, the x-axis represents culture age, and the y-axis is
normalized transcript abundance in log10 scale. Panels in A are from quality controlled time-course data (8 time points)
 while those in B display data from all samples taken (12 time points).

## Slide 6
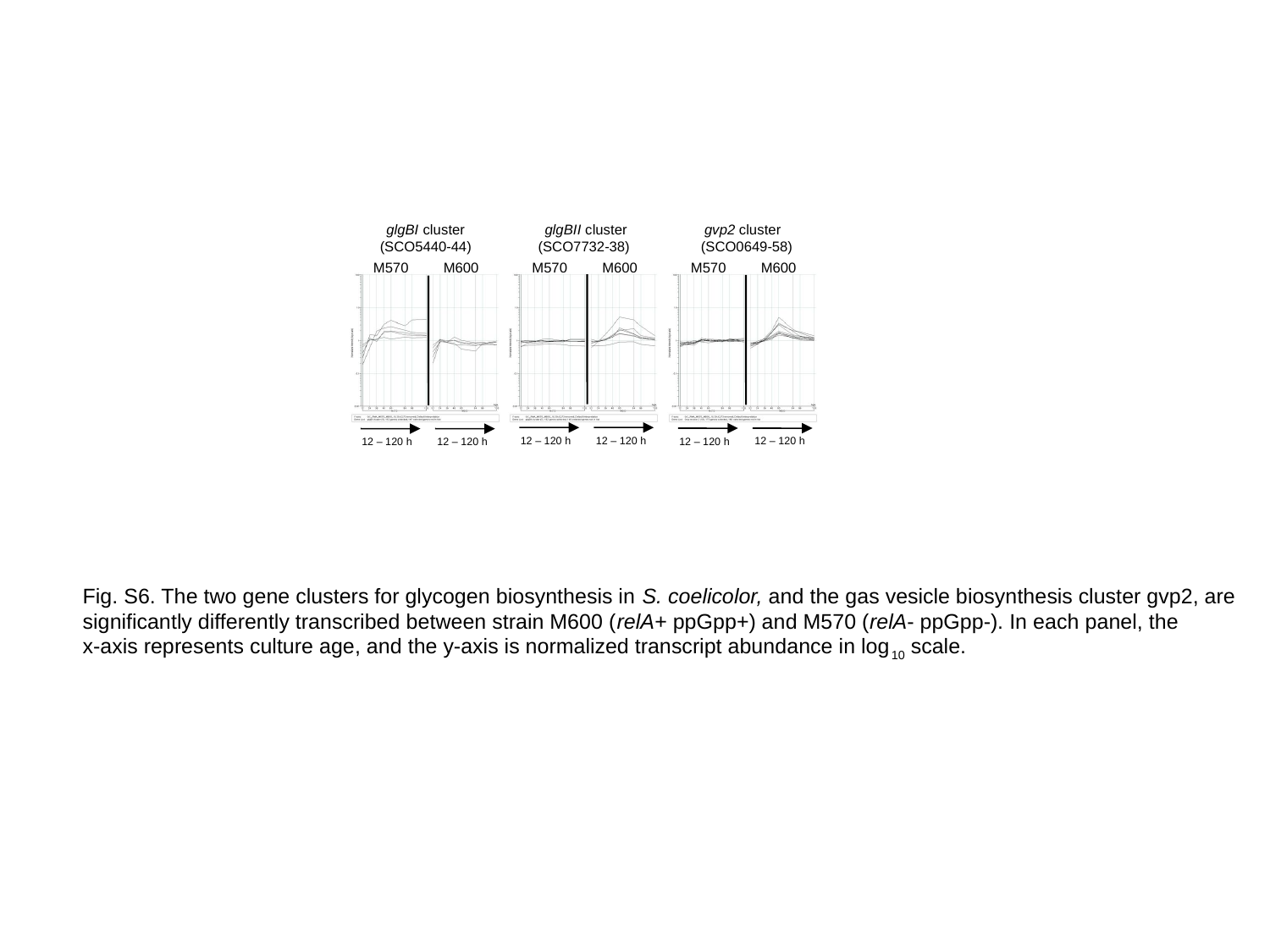

glgBI cluster
(SCO5440-44)
glgBII cluster
(SCO7732-38)
gvp2 cluster
(SCO0649-58)
M570
M600
M570
M600
M570
M600
12 – 120 h
12 – 120 h
12 – 120 h
12 – 120 h
12 – 120 h
12 – 120 h
Fig. S6. The two gene clusters for glycogen biosynthesis in S. coelicolor, and the gas vesicle biosynthesis cluster gvp2, are
significantly differently transcribed between strain M600 (relA+ ppGpp+) and M570 (relA- ppGpp-). In each panel, the
x-axis represents culture age, and the y-axis is normalized transcript abundance in log10 scale.

## Slide 7
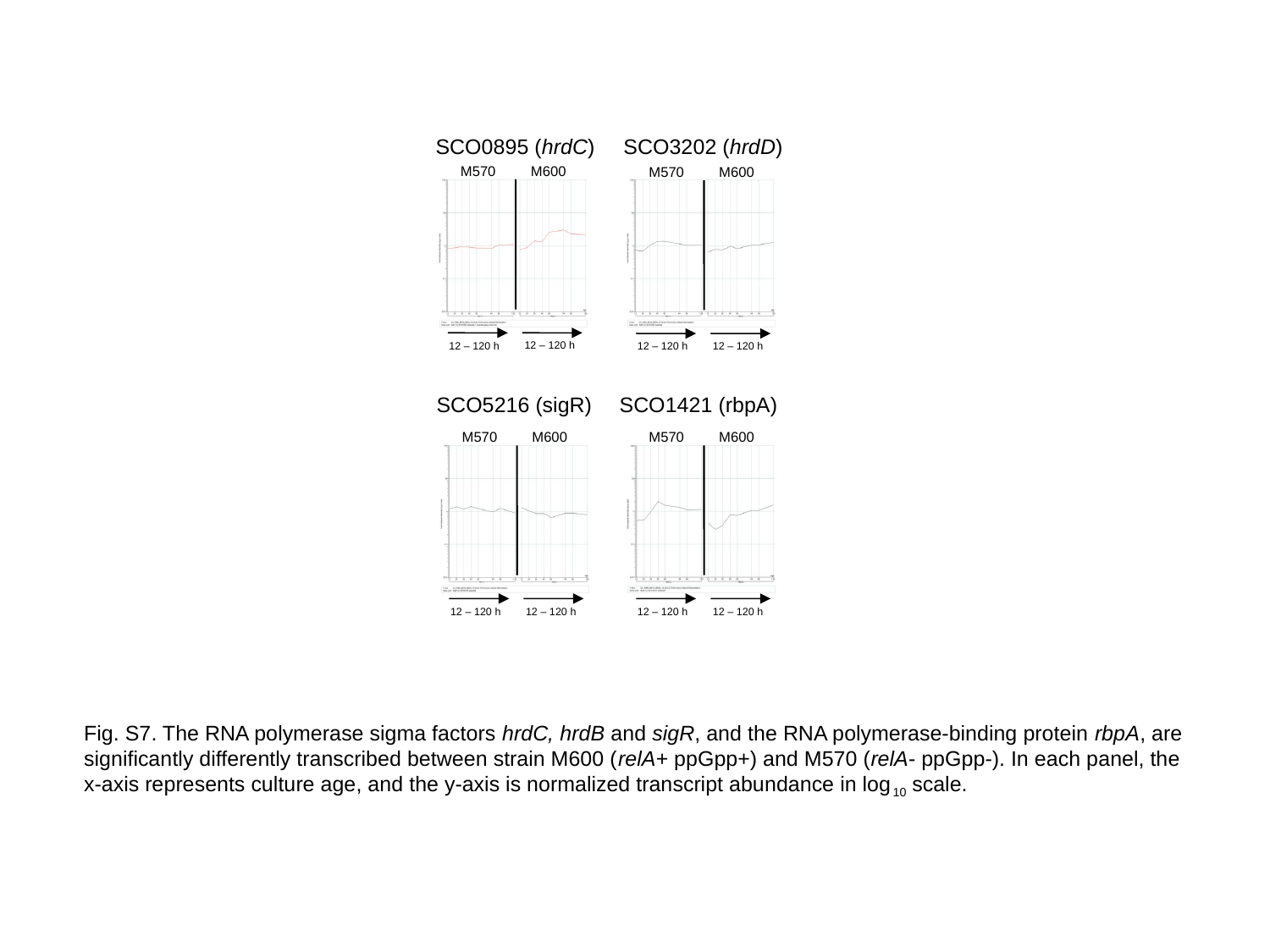

SCO0895 (hrdC)
SCO3202 (hrdD)
M570
M600
M570
M600
12 – 120 h
12 – 120 h
12 – 120 h
12 – 120 h
SCO5216 (sigR)
SCO1421 (rbpA)
M570
M600
M570
M600
12 – 120 h
12 – 120 h
12 – 120 h
12 – 120 h
Fig. S7. The RNA polymerase sigma factors hrdC, hrdB and sigR, and the RNA polymerase-binding protein rbpA, are
significantly differently transcribed between strain M600 (relA+ ppGpp+) and M570 (relA- ppGpp-). In each panel, the
x-axis represents culture age, and the y-axis is normalized transcript abundance in log10 scale.

## Slide 8
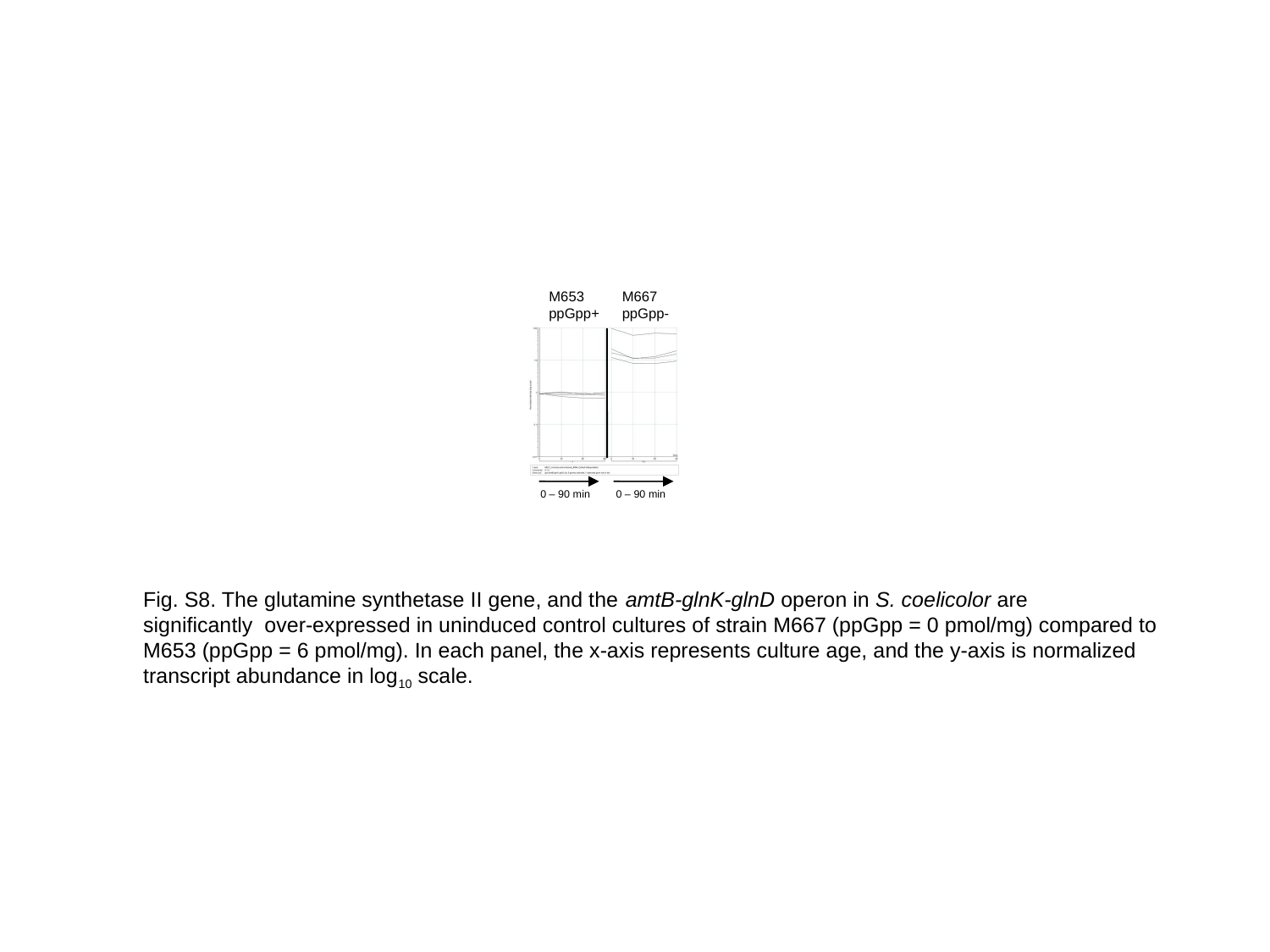

M653
ppGpp+
M667
ppGpp-
0 – 90 min
0 – 90 min
Fig. S8. The glutamine synthetase II gene, and the amtB-glnK-glnD operon in S. coelicolor are
significantly over-expressed in uninduced control cultures of strain M667 (ppGpp = 0 pmol/mg) compared to
M653 (ppGpp = 6 pmol/mg). In each panel, the x-axis represents culture age, and the y-axis is normalized
transcript abundance in log10 scale.
